# Enzymatic Flow Electrolyzer for CO_2_ and Waste Comproportionation to Formate and Its Use in Photocatalytic Alkene Hydrocarboxylation

**DOI:** 10.1002/anie.202515810

**Published:** 2025-09-24

**Authors:** Beverly Q. L. Low, Santiago Rodríguez‐Jiménez, Andrea Rogolino, Samuel J. Cobb, Chen Han, Guilherme Martins, Inês A. C. Pereira, Erwin Reisner

**Affiliations:** ^1^ Yusuf Hamied Department of Chemistry University of Cambridge Lensfield Road Cambridge CB2 1EW UK; ^2^ Instituto de Tecnologia Química e Biológica António Xavier (ITQB NOVA) Universidade NOVA de Lisboa Av. da República Oeiras 2780‐157 Portugal; ^3^ Present address: Department of Chemistry The University of Manchester Oxford Road Manchester M13 9PL UK

**Keywords:** CO_2_ utilization, Electrocatalysis, Enzymes, Photocatalysis, Plastic valorization

## Abstract

Paired electrolysis enables the simultaneous coupling of CO_2_ reduction with anodic waste upcycling to form valuable products. However, achieving selective, efficient, and stable product formation and coupling to downstream valorization remains a challenge. In this study, W‐containing formate dehydrogenase from *Nitratidesulfovibrio vulgaris* Hildenborough is immobilized onto a cathode made from carbon felt coated with porous TiO_2_ and paired with a commercial Ni foam anode to assemble a semiartificial flow electrolyzer for the simultaneous conversion of CO_2_ and waste (plastic and biomass) to the single product formate. The enzymatic flow electrolyzer achieved an initial cell faradaic efficiency toward formate of almost 200%, a maximum CO_2_ conversion yield of 18% and can operate at a low full‐cell voltage of −1.5 V for 122 h, which allows for bias‐free operation with a silicon photovoltaic cell. The aqueous formate produced in the enzymatic electrolyzer was subsequently utilized downstream as a C_1_ building block in the photocatalytic hydrocarboxylation of alkenes, providing a path for the domino valorization of CO_2_ and waste toward bulk and fine chemical synthesis.

## Graphical abstract

A flow electrolyzer employing formate dehydrogenase on a porous TiO_2_‐carbon felt cathode is developed for paired electrolysis of CO_2_ and waste (plastic and biomass) to produce formate. The electrolyzer operates with an initial cell faradaic efficiency toward formate of almost 200% at a low cell voltage of −1.5 V, which also enables bias‐free operation with a commercial solar cell. The electrogenerated formate is used directly for photocatalytic carbon chain extension of styrene to phenylpropanoic acid.

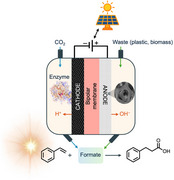

## Introduction

To mitigate the rise in carbonaceous emissions and develop strategies for a circular chemical industry, the development of sustainable solutions to convert CO_2_ into  useful chemical products is crucial.^[^ ^1^, ^2^, ^3^, ^4^ ^]^ Among CO_2_ conversion products, formate, or its protonated form formic acid, presents a useful vector molecule with versatile applications in a net zero fuel and chemical industry. It can be used as i) a fuel directly in fuel cells,^[^ ^5^ ^]^ ii) a liquid energy carrier that can store and release hydrogen,^[^ ^6^ ^]^ iii) a carbon and energy source in biorefineries with bioengineered microorganisms,^[^ ^7^ ^]^ and iv) an intermediate for fine chemical synthesis.^[^ ^8^ ^]^ Specifically, it is also an attractive soluble synthon for carbon chain extension reactions, including alkene and alkyne hydrocarboxylation or the carboxylation of imines.^[^ ^9^, ^10^, ^11^, ^12^, ^13^ ^]^

Currently, 90% of the world's formic acid production is dominated by the methyl formate hydrolysis route, upon which industrial processes (e.g., Kemira–Leonard, BASF) are based on.^[^ ^14^ ^]^ The production volume from the thermochemical Kemira–Leonard process exceeds 100 000 tons per year and involves the liquid‐phase carbonylation of methanol at about 4 MPa and 80 °C in the presence of a basic catalyst to form methyl formate.^[^ ^15^ ^]^ Subsequently, methyl formate is reacted with water at roughly 120 °C and 0.9 MPa to obtain formic acid, which can be distilled to obtain 85 wt% concentration.^[^ ^15^ ^]^ With the high temperatures and pressures employed, the Kemira–Leonard process requires a steam energy of 17.45 MJ kg^−1^ of formic acid.^[^ ^14^ ^]^ Formic acid can also be formed by acidolysis of formate salts, which are derived as an industrial byproduct during the production of polyhydric alcohols, such as the reaction between formaldehyde and acetaldehyde in alkaline conditions to give pentaerythritol and a formate salt in the basic medium.^[^ ^15^, ^16^ ^]^ Formate can also be obtained through pyrolysis, acid hydrolysis (with sulfuric acid), and wet oxidation (with H_2_O_2_) of biomass, but these methods also face the disadvantages of high temperatures (400–600 °C) and O_2_ pressures (1–5 MPa).^[^ ^17^, ^18^ ^]^

In contrast to established industrialized methods, the electrolytic production of formate driven by renewable energy sources (e.g., photovoltaics) provides a greener path to generate formate under mild and sustainable conditions.^[^ ^16^, ^18^ ^]^ The electrocatalytic reduction of CO_2_ to formate provides therefore an entry point for circular formate chemistry.^[^ ^2^, ^3^, ^19^ ^]^ However, the commercial feasibility of CO_2_ to formate electrolysis depends on high selectivity, long‐term operation, and energy‐efficient operation, i.e., running at low voltage.^[^ ^6^, ^16^, ^17^, ^18^ ^]^ While metal catalysts (Pd, Sn, Bi) are known for selective electroreduction of CO_2_ to formate, attaining operation at low overpotential with good stability remains an unmet challenge.^[^ ^20^, ^21^, ^22^, ^23^, ^24^ ^]^ For example, electrolysis in alkaline electrolyte solution suppresses the competing hydrogen evolution reaction (HER), but may result in (bi)carbonate precipitation, flooding, or CO_2_ crossovers, leading to overall low CO_2_ conversion and high cell voltages.^[^ ^16^, ^23^, ^24^, ^25^ ^]^

Enzymes such as formate dehydrogenase (FDH) provide an alternative to utilizing metal catalysts, and some metal‐containing FDHs have been shown to convert CO_2_ and protons to formate with near unity selectivity at marginal overpotentials under neutral pH conditions and ambient temperature.^[^ ^26^, ^27^ ^]^ This has motivated their use as model catalysts with benchmark performance in electrocatalytic, photocatalytic, and photoelectrochemical systems.^[^ ^28^, ^29^, ^30^, ^31^, ^32^, ^33^, ^34^, ^35^, ^36^ ^]^ In particular, the use of select Mo‐ or W‐dependent FDHs (Mo‐ or W‐FDH) allows direct electron transfer (DET) between the enzyme and electrode, and these FDHs have been demonstrated to reduce CO_2_ specifically rather than bicarbonate.^[^ ^37^ ^]^ Electrodes that have been reported for the DET of FDH include metal oxide‐based electrodes as well as functionalized carbon nanotube and graphite electrodes.^[^ ^26^, ^27^, ^29^, ^31^, ^33^, ^34^, ^35^ ^]^ DET‐mode electrolysis for continuous CO_2_ conversion circumvents the use of diffusional mediators employed in existing flow FDH systems, which can be expensive, toxic, kinetically slow, and run the risk of crossovers to the anode compartment.^[^ ^48^, ^49^, ^50^, ^59^, ^60^ ^]^

Apart from CO_2_ reduction, formate can also be obtained from the electrooxidation of waste such as plastics (polyethylene terephthalate, PET) and biomass (glucose from food waste, glycerol from biodiesel manufacturing).^[^ ^38^, ^39^, ^40^, ^41^ ^]^ Earth‐abundant 3d transition metals (Ni, Fe, Co, and Cu) are suitable catalysts for the oxidative valorization of these wastes, in particular through their high oxidation states promoting alcohol oxidation.^[^ ^42^, ^43^ ^]^ Among them, Ni‐based anodes have demonstrated high selectivity toward formate production, owing to their high work function and the in situ formation of active Ni^3+^ sites under alkaline conditions.^[^ ^42^, ^43^, ^44^, ^45^, ^46^, ^47^ ^]^

The comproportionation of CO_2_ and waste can be achieved via paired electrolysis, yielding formate simultaneously from both the reduction and oxidation processes.^[^ ^46^ ^]^ This approach mitigates two waste streams and enables maximum formate production, thereby increasing the economic viability of producing formate sustainably. This process is also energy efficient, with the full‐cell thermodynamics of this paired electrolyzer being favorable compared to traditional electrolyzers that employ water oxidation to O_2_.^[^ ^46^, ^47^, ^48^, ^49^, ^50^ ^]^ Furthermore, by producing a single product (formate) on both electrodes, costly separation steps can be avoided in the event of crossovers between anolyte and catholyte.^[^ ^51^ ^]^ While the dual production of formate by paired electrolysis of CO_2_ reduction and waste oxidation has been reported, existing electrolyzers require a full‐cell voltage close to −2 V in order to achieve high Faradaic efficiency (FE) toward formate.^[^ ^43^, ^48^, ^51^, ^52^, ^53^, ^54^, ^55^ ^]^ The downstream use of formate produced from paired electrolysis has not yet been demonstrated to our knowledge.

**Figure 1:**
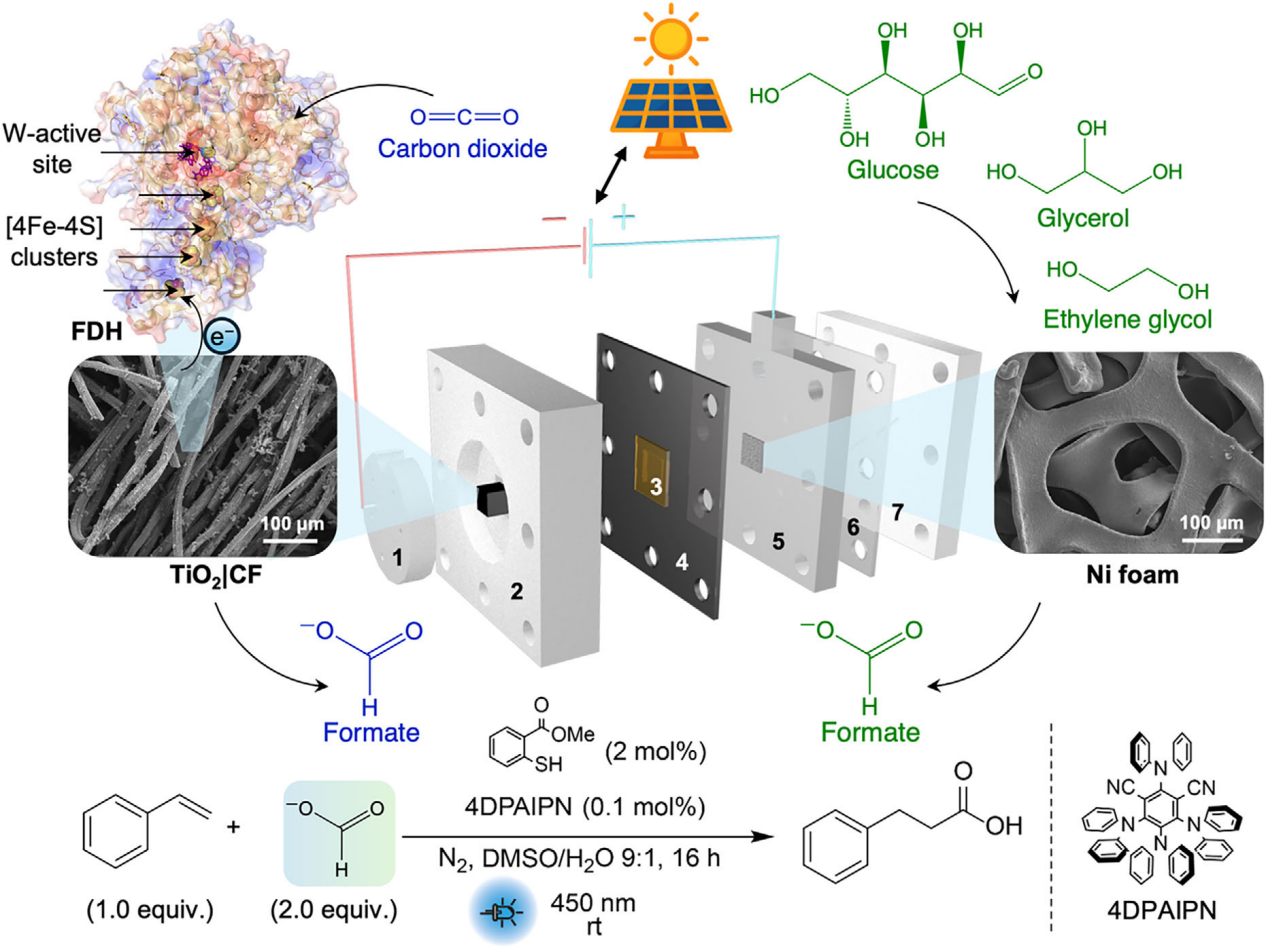
Schematic view of bespoke flow electrolyzer used for the paired electrolyzer coupling an FDH|TiO_2_|CF cathode with a Ni foam anode, separated by a bipolar membrane (BPM). The protein structure of FDH from *Nitratidesulfovibrio vulgaris* Hildenborough (PDB: 6sdv) and the scanning electron microscopy (SEM) images of TiO_2_|CF and Ni foam electrodes are shown. Electrolyzer components are the cathode backplate (labeled 1), cathode flow plate (2), BPM (3), Viton gasket (4), anode flow plate (5), polytetrafluoroethylene (PTFE) gasket (6), and anode backplate (7). The formate produced in the electrolyzer is subsequently utilized for the photocatalytic hydrocarboxylation of styrene to phenylpropanoic acid, as shown in the reaction scheme (concentrations adjusted to 2 equiv. formate from the electrolyte solution). The half‐cell equations and overall cell reaction of CO_2_RR and EGOR are shown in Equations (1), (2), (3).

Herein, we report an enzymatic flow electrolyzer (Figures 1 and S1), which employs a 3D porous cathode composed of W‐FDH from *Nitratidesulfovibrio vulgaris* Hildenborough (*Nv*H) immobilized on TiO_2_‐coated carbon felt (TiO_2_|CF) and a commercial nickel foam as anode. This semiartificial flow electrolyzer can simultaneously electro‐generate formate from CO_2_ and three types of waste‐derived substrates—ethylene glycol (EG) derived from PET plastic waste, glycerol available as a byproduct of biodiesel manufacturing, and glucose from food waste. The paired electrolysis of the CO_2_ reduction reaction (CO_2_RR) and EG oxidation reaction (EGOR) in aqueous electrolyte solution (Equations (1), (2), (3)) was achieved at a low full‐cell voltage of −1.5 V with an initial cell FE of ∼200% toward formate (∼100% FE at both cathode and anode) and a total operating duration of 122 h. The low voltage requirement allowed powering the electrolyzer in bias‐free operation using a Si photovoltaic. The formate produced from electrolysis can subsequently be utilized directly and without purification as a C_1_ synthon for the photocatalytic hydrocarboxylation of alkenes, thereby providing an innovative PV‐powered electrocatalytic–photocatalytic domino strategy to valorize CO_2_ and waste toward sustainable fine chemicals.

(1)

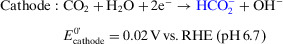



(2)

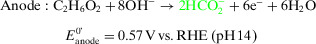



(3)

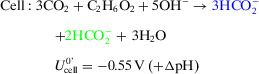




## Results and Discussion

### FDH|TiO_2_|CF Cathode for CO_2_RR

W‐dependent FDH from *Nv*H has been chosen due to its ability to perform the electrochemically reversible interconversion of CO_2_ and protons into formate (*E* ^0’^ = 0.02 V versus RHE, pH 6.7) via DET on porous metal oxide supports and its ability to tolerate small amounts of O_2_.^[^ ^34^, ^56^ ^]^ These properties make it a unique catalyst to achieve selective formate production in aqueous solution at a marginal overpotential, which makes this FDH an ideal choice to assemble a low‐voltage electrolyzer.

To enable long‐term CO_2_RR in a flow electrolyzer with suitable current densities, robust immobilization of the electroactive FDH is required on a conductive and high surface area scaffold. Indium tin oxide (ITO)‐coated carbon felt electrodes have been recently reported as a suitable electrode scaffold for FDH immobilization,^[^ ^57^ ^]^ but ITO is susceptible to degradation at reducing potentials (<−0.4 V versus RHE) and will thus not be stable for operation in an electrolyzer.^[^ ^58^ ^]^ Porous TiO_2_ on conducting glass substrates has been shown to immobilize FDH via non‐covalent interactions at high loading and is stable toward more reducing potentials.^[^ ^34^, ^59^ ^]^ Therefore, we developed a TiO_2_|CF electrode by sintering TiO_2_ nanoparticles (∼25 nm, Figure S2) on commercially available carbon felt cuboids (0.5 × 0.5 × 0.64 cm^3^), which provides a 3D scaffold for stable FDH immobilization in the enzymatic electrolyzer. TiO_2_|CF was prepared by drop‐casting a TiO_2_ suspension (10 mg mL^−1^) onto CF followed by annealing at 400 °C. The TiO_2_|CF electrode was characterized by SEM and energy‐dispersive X‐ray spectroscopy (EDX) (Figures 1 and S2, S3), which confirms the formation of TiO_2_ nanoparticles on the carbon felt substrate. FDH was loaded onto TiO_2_|CF (FDH|TiO_2_|CF) by drop‐casting the enzyme solution under inert conditions.

**Figure 2:**
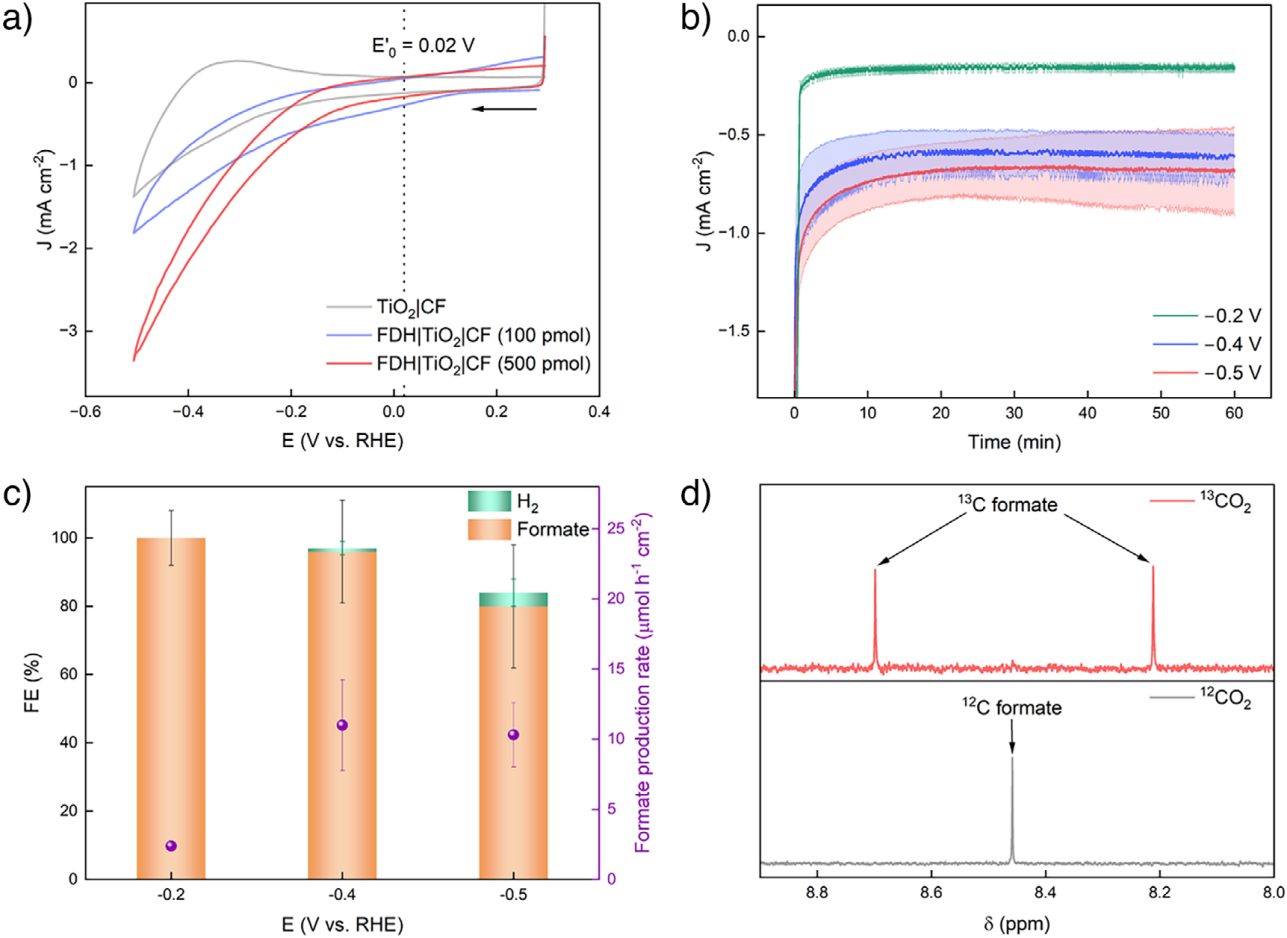
a) PFV of FDH|TiO_2_|CF (red, 500 pmol FDH; blue, 100 pmol FDH) with the background scan (grey) of bare TiO_2_|CF electrode at a scan rate of 10 mV s^−1^. b) CPE of FDH|TiO_2_|CF cathode at different applied potentials (V versus RHE), and c) the corresponding FE of products and rate of formate production. Experiments were conducted in triplicates, where the mean is shown with standard deviation represented by the shaded region or bars. d) ^1^H NMR peaks of the ^13^C and ^12^C‐labeled formate corresponding to ^13^CO_2_ or ^12^CO_2_ used. Conditions: 100 pmol of FDH loading (except red trace in (a)), Ag/AgCl and Pt wire used as the RE and CE, respectively, 100 mM NaHCO_3_ with 50 mM KCl (5 mL) used as the electrolyte and purged with CO_2_ for 20 min prior to measurement (pH 6.7), ambient temperature (25 °C). For the isotopic labeling experiment, the electrolyte (100 mM NaH^13^CO_3_ with 50 mM KCl) was purged with N_2_ for 10 min and evacuated prior to the introduction of ^13^CO_2_.

The electrochemical characterization of the prepared electrodes (TiO_2_|CF and FDH|TiO_2_|CF) was conducted in a three‐electrode configuration (H‐cell) with a Ag/AgCl reference electrode (RE), Pt counter electrode (CE) and CO_2_‐saturated aqueous bicarbonate buffer solution (100 mM NaHCO_3_ with 50 mM KCl, pH 6.7) under ambient conditions (25 °C). The cyclic voltammetry (CV) scan (Figure 2a) of the bare TiO_2_|CF electrode (grey) shows the expected response of TiO_2_, where a reductive current is observed with an onset of −0.1 V versus the reversible hydrogen electrode (RHE),^[^ ^31^ ^]^ followed by an anodic peak (−0.3 V versus RHE), corresponding to the charging and discharging of the conduction band of TiO_2_.^[^ ^60^ ^]^ This electrochemical response indicates that there is good electrical connection and charge transport established between the carbon felt and the sintered TiO_2_ nanoparticles.

When FDH (500 pmol) was dropcast, the protein film voltammetry (PFV) of the resulting FDH|TiO_2_|CF cathode (red, Figure 2a) shows a prominent catalytic wave with an onset potential close to the TiO_2_ conduction band and thermodynamic potential of CO_2_ to formate conversion, indicating the efficient interfacial electron transfer by the [4Fe–4S] relays and catalytic activity at the W‐active site of FDH.^[^ ^34^ ^]^ The absence of the oxidative peak at −0.3 V versus RHE further supports the effective DET from TiO_2_ to FDH and consumption of conduction band electrons by catalytic CO_2_RR. Lowering the FDH loading (100 pmol dropcast; blue, Figure 2a) decreases the current density from −3.3 mA cm^−2^ to −1.8 mA cm^−2^ at −0.5 V versus RHE.

Controlled potential electrolysis (CPE) of the FDH|TiO_2_|CF electrode (100 pmol FDH loading) was then measured at applied potentials of −0.2, −0.4, and −0.5 V versus RHE (Figure 2b,c). Similar to previous FDH‐loaded electrodes reported,^[^ ^34^, ^57^, ^59^ ^]^ the FDH|TiO_2_|CF electrodes attain ∼100% FE toward formate at low overpotential (−0.2 V versus RHE). FE toward formate decreased to 80% when a more reducing potential (−0.5 V versus RHE) was applied (Figure 2c), with the HER becoming a competing side reaction. This is consistent with the PFV scan of the FDH|TiO_2_|CF cathode after CPE (Figure S4), where unlike the PFV scan prior to CPE, the anodic wave (−0.3 V versus RHE) reappears, suggesting that electrons discharged from the conduction band of TiO_2_ instead of transferring to FDH, hence the decrease in FE toward formate.^[^ ^60^ ^]^ The post‐reaction SEM and EDX characterization shows minimal changes to the morphology of the TiO_2_|CF electrode (Figures S5 and S6). The control experiments with a bare TiO_2_|CF electrode produced only hydrogen (FE = 92%) with no formate being detectable (Figure S7). Isotopic labeling experiments with FDH|TiO_2_|CF electrodes using ^13^CO_2_ and aqueous NaH^13^CO_3_ confirmed CO_2_ as the sole carbon source, with ^13^C‐labeled formate being detected by ^1^H NMR spectroscopy (8.46 ppm, doublet, *J*_C‐H_ = 196 Hz, Figure 2d).

Although FDH‐loaded inverse opal (IO)‐TiO_2_ electrodes have previously maintained a high FE (96%) toward formate at highly reducing potentials (−0.53 V versus RHE),^[^ ^31^ ^]^ the brittle IO‐TiO_2_ thin films (40 µm film thickness) cannot be employed in a flow electrolyzer as they are limited in stability under those conditions during hour‐long operation. Our 3D porous matrix of TiO_2_‐coated CF provides a robust electrode, which enables higher FDH loadings (500 pmol) and longer‐term CO_2_ reduction (24 h, Figure S8), hence providing a promising strategy for the application of FDH in a flow electrolyzer. Leveraging the low overpotentials required by the FDH|TiO_2_|CF cathode (−0.2 V versus RHE), a CO_2_ flow electrolyzer device with low full‐cell voltage can be achieved.

### Ni Foam for Waste Oxidation

In existing CO_2_ electrolysis systems, the cathodic CO_2_RR is often paired with the anodic oxygen evolution reaction (OER).^[^ ^53^, ^61^, ^62^, ^63^ ^]^ However, OER requires a highly positive thermodynamic potential (1.23 V versus RHE), possesses sluggish kinetics, and gives rise to a product of low economic value (O_2_).^[^ ^64^ ^]^ The oxidation of small organic molecules is significantly less thermodynamically demanding, and by coupling it to CO_2_RR we can yield another valuable product of industrial interest.^[^ ^46^, ^47^ ^]^ Furthermore, forming the same product (formate) on both the anode and cathode streams would be advantageous in paired electrolysis as this maximizes overall formate production and eliminates issues from product crossover.

To achieve high overall formate selectivity in the paired electrolyzer, the FDH|TiO_2_|CF cathode should be paired with an anode capable of substrate oxidation to formate with high selectivity. Ni‐based electrodes have been previously reported to selectively oxidize substrates such as EG, glycerol, and glucose to formate.^[^ ^43^, ^54^, ^55^, ^65^ ^]^ These substrates are of particular interest as they can be sourced from ubiquitous wastes, enabling co‐valorization of both cathodic and anodic reactions (Equations (1), (2), (3)). EG can be viably obtained from pretreatment of PET plastic wastes,^[^ ^66^ ^]^ which also simultaneously addresses the problem of environmental pollution.^[^ ^67^ ^]^ Glycerol is a byproduct that accounts for 10 wt% of biodiesel and soap manufacturing and due to its excess supply and low market value, glycerol valorization has also become of great interest.^[^ ^68^, ^69^ ^]^ Glucose is another feasible anodic substrate, which can be obtained from processing biomass wastes, such as cellulose or food waste.^[^ ^70^, ^71^, ^72^, ^73^, ^74^, ^75^, ^76^, ^77^, ^78^ ^]^ 1.3 billion tons of food waste are generated annually, amongst which some (e.g., apple) mainly consists of water‐soluble sugars (fructose, glucose, and sucrose). Hence, obtaining glucose from food waste is simple and more cost‐effective compared to the pretreatment of cellulose.

**Figure 3:**
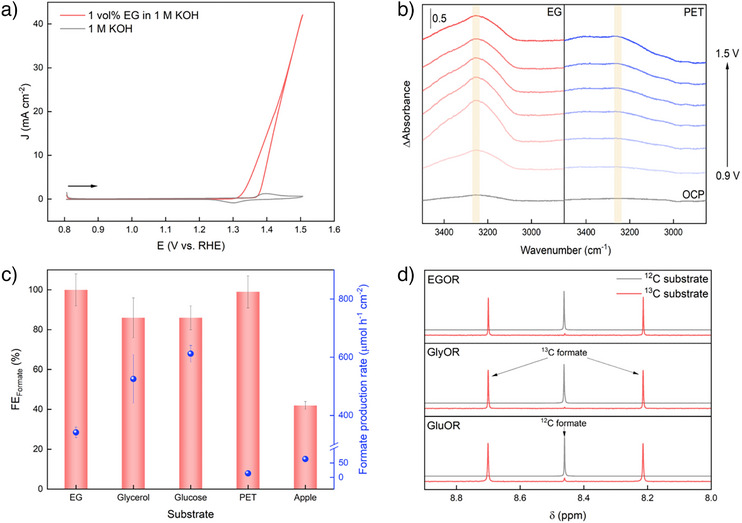
a) CV scan of Ni foam in the absence (grey) and presence (red) of EG, at a scan rate of 10 mV s^−1^. b) In situ IR spectra measured with increasing applied potentials (V versus RHE) in a solution of 0.1 vol.% EG in 0.1 M KOH (red) or 10 times diluted pretreated PET solution (blue). ΔAbsorbance was calculated by subtracting the background scan measured without applied potential (OCP). Ni ink painted on Au was used as the working electrode (WE). c) FE toward formate and the rate of formate production for 1 h of CPE over Ni foam in the presence of different substrates, performed in triplicates (mean is shown with standard deviation). d) Control experiments with ^13^C‐labeled substrates for EGOR, GlyOR, and GluOR. Conditions (c,d): 1.5 V versus RHE applied potential, Ag/AgCl and Pt wire were used as RE and CE respectively, 1 vol.% of substrate (or 87 mM of glucose or 10 mM of ^13^C‐labeled substrates) in 1 M KOH (pH 14, 5 mL) or as‐prepared PET and apple solutions were used as electrolyte, ambient temperature (25 °C).

Commercial Ni foam of 95% porosity with an average hole diameter of 0.25 mm was compressed into paper‐sheet thickness (∼0.3 mm) and employed without further modification for the oxidation of EG and glucose as model substrates and glycerol, pretreated PET and apple solutions as real‐world‐derived substrates. The electrochemical behavior of the Ni foam anode was preliminarily investigated by CV in aqueous KOH (1 M, pH ∼14) electrolyte solution (Figure 3a). The redox waves at *E*_p/2_ = 1.32 V versus RHE can be attributed to the reversible Ni^2+^/Ni^3+^ redox couple.^[^ ^79^ ^]^ In the presence of EG (1 vol.%, 180 mM), the anodic peak is replaced by a catalytic curve with an onset at ∼1.3 V versus RHE, suggesting that the oxidation of EG takes place at the Ni^3+^ sites.^[^ ^43^ ^]^ CPE in the presence of EG shows that the Ni foam can attain a current density of around 40 mA cm^−2^ at 1.5 V versus RHE (overpotential of 0.93 V, Figure S9a). This current density obtained by the unmodified commercial nickel foam is also comparable with other Ni‐based anodes reported for EG oxidation, such as NiSe_2_ (40–95 mA cm^−2^ at 1.6 V versus RHE, 1 M EG) as well as CuO@Ni(OH)_2_ (50 mA cm^−2^ at 1.36 V versus RHE, 100 mM EG).^[^ ^43^, ^79^ ^]^

The current density drastically decreases when EG is replaced by the real‐world EG solution obtained by alkaline pretreatment of a PET bottle (Figure S9a,b) in aqueous KOH (1 M) at 80 °C for 72 h under stirring. Given that the concentration of EG in the pretreated PET solution was also ∼1 vol.%, the drop in current density may be attributed to the presence of terephthalate (TPA, Figure S9c), which competitively adsorbs on the catalytic sites of the Ni anode, inhibiting the adsorption of EG for conversion.^[^ ^35^ ^]^ This was also confirmed with the exogenous addition of TPA to a solution of pure EG, mimicking the pretreated PET solution, which indeed resulted in a decrease in current density (Figure S9a). Nonetheless, the presence of TPA did not affect the near‐unity selectivity of EG conversion to formate and ∼100% FE toward formate is observed for the oxidation of PET solution (Figure S9d). Exclusion control experiments confirmed that formate was not produced in the absence of EG.

To understand the difference in employing the pure EG substrate and real‐world PET waste, in situ IR spectroscopy was measured over Ni powder in the presence of EG or PET solutions (Figures 3b and S10a). When a solution of 0.1 vol.% EG in KOH (0.1 M) was measured, a band at 3255 cm^−1^ is observed, which can be attributed to the overlapping contributions of surface‐bound OH groups and EG, supporting the adsorption of EG onto the Ni powder (Figure S10b). The adsorption of EG increased with higher applied potentials (Figure 3b), consistent with the increase in current densities observed during CV. Meanwhile, in the presence of the PET solution, the OH‐vibration at 3255 cm^−1^ is diminished and instead new peaks are observed at 1680 cm^−1^, corresponding to the C═O stretching vibration of the −COO^−^ group in TPA (Figure S10c),^[^ ^80^ ^]^ indicating the adsorption of TPA instead. Furthermore, the EG adsorption (3255 cm^−1^) was also lowered upon addition of TPA into a solution of EG in KOH (0.1 M), mimicking the PET solution (Figure S10d). Hence, the difference in current densities observed for the oxidation of EG and PET solutions can be attributed to the difference in EG adsorption, most reasonably caused by the coordination of TPA to the surface of the Ni electrode. Removal of TPA by further processing can alleviate the effect of the poisoning, which also enables the use of TPA to produce new virgin plastics.

Similar to the oxidation of EG and PET solution, the oxidation of glycerol (GlyOR, 1 vol.%, 140 mM) and glucose (GluOR, 78 mg, 87 mM) at 1.5 V versus RHE also shows high FE (>80%) toward formate (Figures 3c and S11, S12). Other products from GlyOR also include glycolate (FE = 5 ± 1%) and glycerate (FE = 3 ± 1%), giving rise to FE_total _= 94 ± 9% (Figure S11b).^[^ ^48^ ^]^ For the oxidation of real‐world glucose obtained from apples (Apple‐OR), store‐bought Royal Gala apples were peeled, cut into small pieces and pretreated by soaking in deionized water at 80 °C for 24 h. Compared to GluOR, Apple‐OR results in lower current density and the FE toward formate (Figure S12) decreases from 86 ± 6% (GluOR) to 42 ± 2% (Apple‐OR). This is likely due to a lower concentration of glucose (∼7 mM) present in the pretreated apple solution as well as the presence of other constituents (organic acids, amino acids, and phenolic compounds) that likely interfered with the adsorption and oxidation of glucose.^[^ ^73^, ^76^, ^81^ ^]^ Isotopic labeling experiments using ^13^C‐labeled EG, glycerol, and glucose show the formation of ^13^C‐labeled formate in ^1^H NMR spectroscopy, confirming that the source of formate production are the respective substrates (8.46 ppm, doublet, *J*_C‐H_ = 196 Hz, Figure 3d).

Compared to other reported Ni‐based anodes for small organic molecule (EG, glycerol, and glucose) oxidation, the use of a commercial Ni foam anode used in this study also achieved comparable FE toward formate (>80%) and current densities (<50 mA cm^−2^ at 1.5 V versus RHE).^[^ ^43^, ^54^, ^65^, ^79^ ^]^ The Ni foam also shows excellent selectivity (∼100%) toward formate for the oxidation of EG and PET solution, exceeding those of previous reports (≤90%).^[^ ^43^, ^79^, ^82^ ^]^ Furthermore, we applied the Ni foam anode to a scope of substrates, including real‐world‐derived waste, demonstrating the versatile and selective formate production (>80%) via different anodic reactions.

### Paired Flow Electrolysis

To realize an enzymatic flow electrolyzer for selective and durable formate production, the FDH|TiO_2_|CF cathode and Ni foam anode were paired in a custom‐designed two‐electrode flow electrolyzer, consisting of a 3D‐printed cathode compartment and a stainless‐steel anode compartment (Figure S13). Unlike the majority of commercially available electrolyzers catered for heterogenous catalysts on gas diffusion electrodes (GDE), this cell was designed with a unique flow path through the cathode, optimized for flowing CO_2_‐saturated catholyte to FDH with the aim of maintaining a suitable local microenvironment (i.e., pH ∼7) for maximum enzyme activity.^[^ ^83^ ^]^ The flow inlet on the cathode backplate allows the CO_2_‐saturated catholyte to first flow through the FDH‐loaded carbon felt, then to the membrane sandwiched between the cathode and anode compartments, before exiting through the outlet on the cathode flow plate. The activity of enzymes is optimal around neutral pH, whereas the Ni foam anode requires basic conditions for high performance. Thus, a BPM was chosen to allow for the pH difference and minimize crossovers between the catholyte and anolyte.

**Figure 4:**
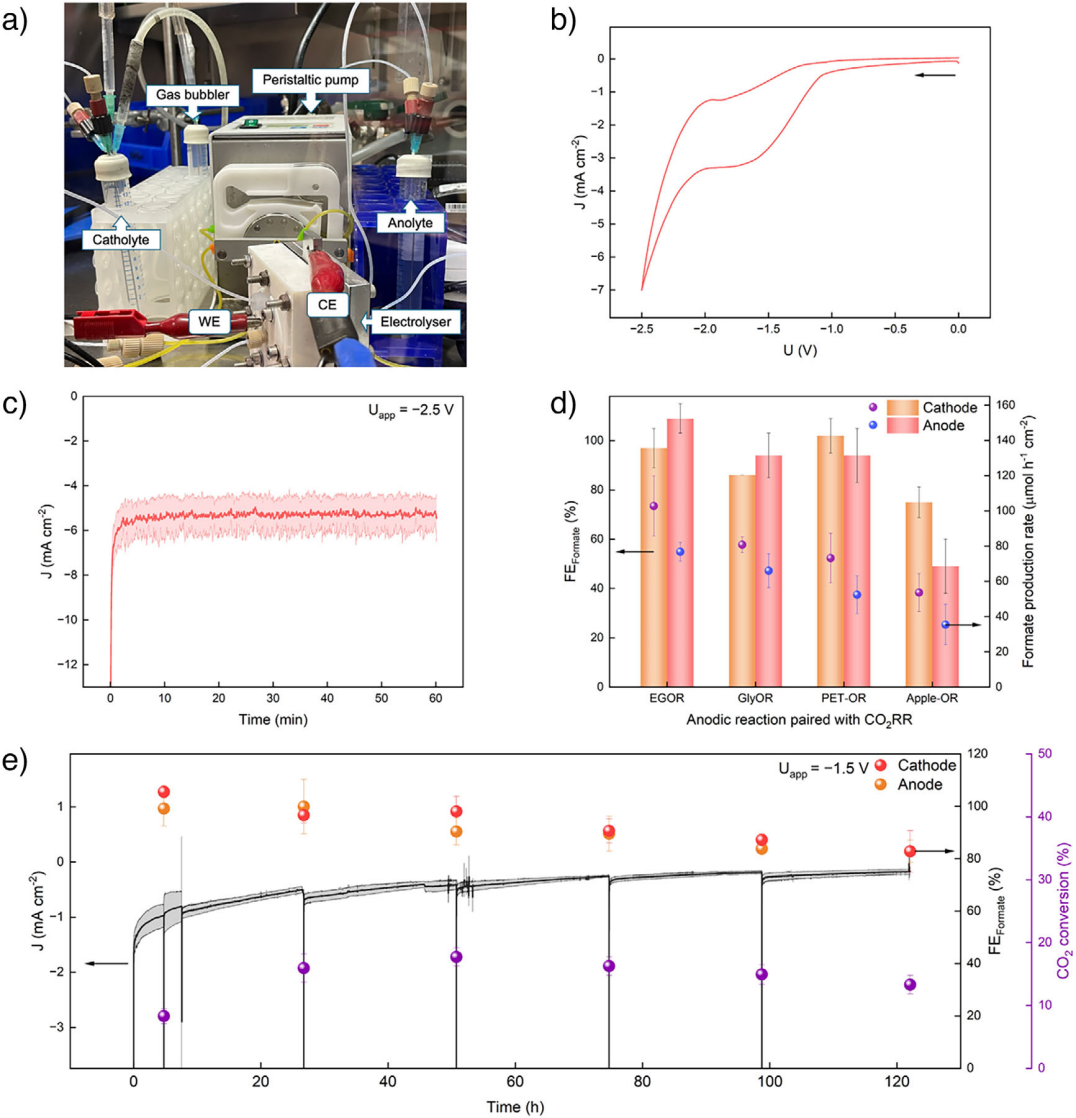
a) Experimental setup of the flow electrolyzer employed for paired electrolysis of FDH|TiO_2_|CF as cathode (WE) and Ni foam as anode (CE). b) CV and c) CPE of paired electrolysis of CO_2_ and EG. d) FE toward formate and formate production rate for cathodic and anodic reaction over 1 h of paired electrolysis, using different anodic substrates. e) Long‐term paired electrolysis of CO_2_ and EG. Experiments were conducted in triplicates, where the mean is shown with standard deviation represented by the shaded region or bars. Conditions: 500 pmol FDH loading, ambient temperature (25 °C), catholyte (100 mM NaHCO_3_ with 50 mM KCl, 10 mL), and anolyte (1 vol.% substrate in 1 M KOH or as‐prepared PET and apple solutions, 10 mL) were constantly flowing through the respective compartments at a rate of 0.5 mL min^−1^. A voltage of −1.5 or −2.5 V was applied, and a BPM was used.

Leveraging the 3D porous matrix of the TiO_2_|CF electrode and to optimize current density, a high FDH loading (500 pmol) was used for measurements in the flow electrolyzer. The flow electrolyzer was set up as shown (Figure 4a) in a N_2_‐filled glovebox at ambient temperature (25 °C), and the electrolytes were continuously flowed through the respective compartments at a rate of 0.5 mL min^−1^. When CO_2_RR was paired with EGOR, the CV curve (Figure 4b) shows an onset voltage of about −1.0 V and reaches a current density of −7.0 mA cm^−2^ at −2.5 V. The broad wave observed at around −1.0 to −2.0 V is characteristic of the charging of the conduction band of TiO_2_ to initiate CO_2_RR with FDH, and a similar wave is observed in the CV curve of bare TiO_2_|CF, i.e., without FDH (Figure S14).^[^ ^60^ ^]^

Given the pH difference of both the electrolyte solutions (ΔpH ∼7), a chemical bias of about 0.4 V should also be accounted for in addition to the electrochemical bias. Apart from driving the half reactions, the applied voltage is also required to overcome the resistances posed by the electrolyzer, such as the voltage contribution from water dissociation at the BPM interface under reverse bias.^[^ ^84^ ^]^ The presence of both catholyte and anolyte also increases the overall internal resistance, as opposed to typical electrolyzers, which use GDEs as cathodes.^[^ ^85^ ^]^ As such, an applied voltage of −2.5 V was initially employed for paired electrolysis. The current density of the paired electrolysis between CO_2_RR and EGOR maintained at −5 mA cm^−2^ for 1 h (Figure 4c), giving rise to around 100% FE toward formate at each electrode (Figure 4d).

Upon confirming the selective dual formate production in the flow electrolyzer, CO_2_RR on the FDH|TiO_2_|CF cathode was subsequently paired with the other waste oxidation reactions (Figure S15). A high cell FE (≥180%) toward formate was achieved when CO_2_RR was paired with GlyOR and PET‐OR (Figure 4d). In contrast, the FE toward formate was lowered when CO_2_RR was paired with Apple‐OR. The lower selectivity toward formate on the anode is likely due to inhibition by other constituents in the apple solution, as observed earlier. Meanwhile, the lower cathodic FE toward formate could be attributed to the presence of ethanol (∼2 mM), which likely crossed over from the anode compartment (fermentation of the apple solution) and may have partially inhibited the activity of FDH (Figure S16).

Leveraging the low overpotential that FDH requires for CO_2_ reduction, the flow electrolyzer was also operated at a low full‐cell voltage of −1.5 V (Figures 4e and S17). To the best of our knowledge, this semiartificial electrolyzer employs the lowest reported full‐cell voltage that can achieve highly selective paired electrolysis to the single product formate (Table S1). The advantage of using a low full‐cell voltage is its energy efficiency and the ease of powering the electrolyzer by renewable energy (e.g., solar and wind energy). To demonstrate this, we have coupled a commercial Si photovoltaic (PV) to the paired electrolyzer under bias‐free conditions (Figure S18a). Under white LED irradiation (10 mW cm^−2^) in a glovebox, the Si PV could drive the electrolyzer to attain an initial current density of −1.5 mA cm^−2^ (corresponding to cell voltage of ∼1.25 V, Figure S18b).

Another advantage of this enzymatic flow electrolyzer is the high selectivity toward formate. During the paired electrolysis of CO_2_ and EG at −1.5 V full‐cell voltage (Figure 4e), dual formate production was achieved with an initial cell FE of ∼200%, which maintained above 160% even after 122 h of operation, highlighting the high selectivity of FDH and Ni foam toward formate production. Furthermore, the flow system enabled the long‐term operation (122 h) of an FDH‐loaded electrode (Table S2) with CO_2_ conversion yields up to 18%. To enable the calculation of CO_2_ conversion for the enzymatic flow electrolyzer (Table S3), the amount of formate produced was quantified at periodic time intervals and the catholyte was re‐saturated with CO_2_. The small increase in current density at each sampling point was likely a result of the intermittent CO_2_ saturation. Unlike typical CO_2_‐paired electrolyzers that flow CO_2_ continuously, the CO_2_‐saturated electrolyte was continuously recirculated through the FDH|TiO_2_|CF cathode to ensure greater CO_2_ utilization and achieve higher formate concentrations (Figure S19).

### Photocatalytic Valorization of Formate

Finally, to illustrate the use of formate as an attractive intermediate molecule for chemical synthesis, HCOO^−^ produced from CO_2_ and EG via flow electrolysis (Figure 4e) was directly utilized as a C_1_ building block for carbon chain extension. Leveraging the benefits of flow electrolysis, electrolytically generated formate could be obtained in sufficiently high concentrations (15 ± 2.5 mM and 23.3 ± 3.8 mM for the anolyte and catholyte, respectively, Figure S19) to be used as a reactant for organic synthesis at µmol scale. Formate is an ideal C_1_ synthon for carbon chain extension reactions, with the photoinitiated radical hydrocarboxylation of alkenes representing a sustainable route for the introduction of carboxylate units under mild conditions.^[^ ^9^, ^10^, ^11^, ^12^, ^86^ ^]^ In particular, in the presence of thiols as radical initiators and a photosensitizer, formate undergoes hydrogen atom abstraction to generate CO_2_^•−^ radical anions, which in turn were shown to convert activated alkenes to carboxylic acids.^[^ ^9^ ^]^ The addition of formate into functionalized hydrocarbons also represents an excellent strategy for in situ utilization of a difficult to isolate aqueous salt into organic molecules that can be obtained with high purity.

Aqueous formate recovered after paired electrolysis of CO_2_ and EG (Figures 4e and S19) was used without further separation or purification in the photocatalytic, thiol‐induced conversion of styrene derivatives to the corresponding substituted phenylpropanoic acids (also known as hydrocinnamic acids) under mild conditions.^[^ ^9^ ^]^ Phenylpropanoic acids and their derivatives find use as fragrances in the cosmetics industry and as food additives.^[^ ^87^ ^]^ The reactions were conducted at room temperature under inert atmosphere for 16 h in a mixture of 10 vol.% aqueous formate and 90 vol.% DMSO (1 mL total volume in a 11 mL vial), blue light irradiation (*λ* = 450 nm, 80 ± 13 mW), in the presence of 2,4,5,6‐tetrakis(diphenylamino)isophthalonitrile (4DPAIPN, *λ*_max_ = 470 nm, Figure S20) as photosensitizer and methyl thiosalycilate as radical initiator (Figure 1 and Table 1). Products were detected and quantified by high‐performance liquid chromatography (HPLC).

**Table 1:** Photocatalytic styrene hydrocarboxylation using electrogenerated formate obtained after 122 h of electrolysis at −1.5 V applied voltage

Entry	Formate solution	Styrene (µmol)	Irradiation^a)^	Phenylpropanoic acid yield (%)^b)^	Styrene conversion (%)
1	Catholyte	0.5	450 nm	73 ± 4	>99
2	Anolyte^c)^	0.5	450 nm	66 ± 6	>99
3	Catholyte:anolyte 1:1^c)^, ^d)^	0.5	450 nm	100 ± 3	>99
4	Catholyte^d)^	5	450 nm	99 ± 8	>99
5	Catholyte	5	White LED	38 ± 3	>99

^a)^ 450 nm LED: 80 ± 13 mW. White LED: ≈100 mW cm^−2^.

^b)^ Error bars are calculated as the standard deviations of triplicates.

^c)^ The anolyte was neutralized to pH 6–7 with 1 M HCl.

^d)^ Solutions were concentrated by evaporation under reduced pressure.

**Figure 5:**
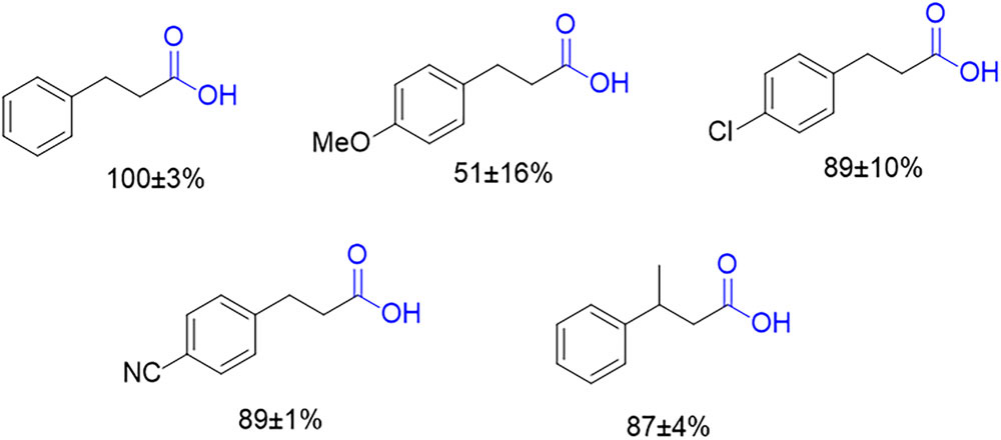
Photocatalytic reaction scope showing products using different styrene derivatives as substrates, with the reaction equation as shown in Figure 1. Reactions were conducted with a 1:1 mixture of catholyte and anolyte. The anolyte was neutralized to pH 6–7 with 1 M aqueous HCl. Error bars are calculated as the standard deviations of triplicates.

After optimization of the reaction conditions with exogenous aqueous potassium formate (Table S4), reactions were performed with aqueous formate solutions from the electrolyte (Table 1) using styrene as a model substrate. The formate‐containing electrolyte solution (catholyte, anolyte, or a mixture) was diluted 10 times in DMSO, i.e., DMSO/H_2_O volume ratio of 9:1. Aqueous formate from both the catholyte (entry 1, Table 1) and the anolyte (entry 2) successfully converted styrene into phenylpropanoic acid with good yields (73 ± 4% and 66 ± 6%, respectively), indicating that additional components of the electrolyte (NaHCO_3_, KCl, and EG) did not significantly interfere with the reaction. Due to the highly alkaline conditions required for EG electrooxidation, the anolyte was neutralized with 1 M HCl to pH 6–7 before addition to the reaction mixture. The catholyte could be used without further treatment. A 1:1 mixture of catholyte and anolyte (followed by dilution in DMSO) resulted in quantitative conversion of styrene into phenylpropanoic acid (entry 3), demonstrating the benefit of formate comproportionation, i.e., single product from both cathodic and anodic streams. As the concentration of formate solutions used in entries 3 and 4 were <10 mM, the solutions were concentrated by solvent evaporation under reduced pressure, which likely resulted in the higher yields. The amount of formate produced by prolonged electrolysis (122 h, Figure S19) was also sufficient to scale the reaction up to 5 µmol using a concentrated electrolyte solution (entry 4). Moreover, styrene conversion also proceeded under white light irradiation, although resulting in lower yields within the same reaction time (entry 5, 38 ± 3% phenylpropanoic acid yield). The protocol with electrogenerated formate also exhibited some functional group tolerance (Figure 5).

**Figure 6:**
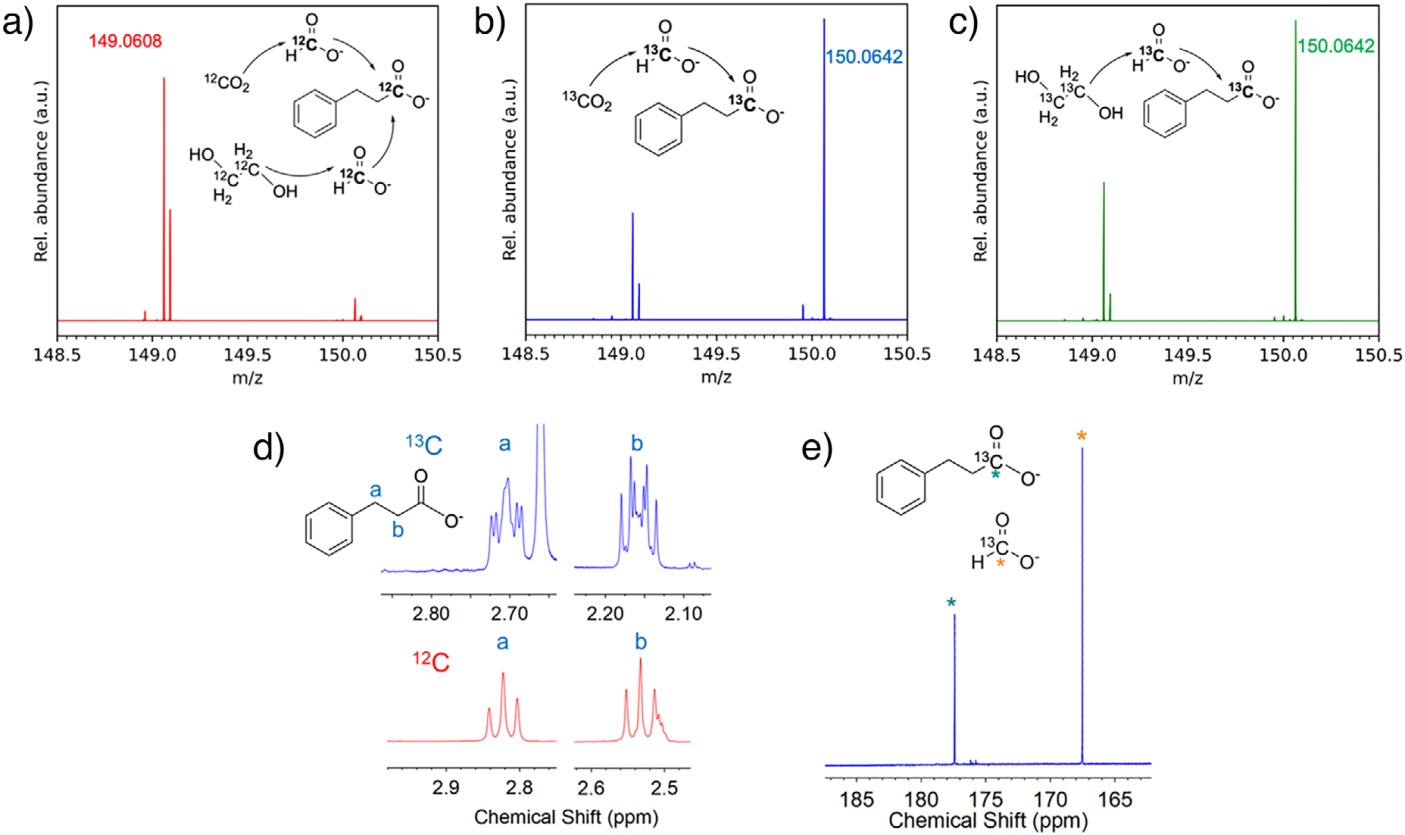
Mass spectra of phenylpropanoic acid obtained by photocatalytic hydrocarboxylation of styrene with electrogenerated formate from a) a 1:1 mixture of catholyte and anolyte from CO_2_ and EG with natural isotopic abundance, b) the catholyte when purged with ^13^CO_2_, c) the anolyte when prepared with EG‐^13^C_2_. d) ^1^H NMR (DMSO, 400 MHz) spectrum of phenylpropanoic acid produced with formate sourced from EG‐^13^C_2_ in the anolyte (blue trace) and of standard phenylpropanoate with natural abundance (red trace). e) ^13^C NMR (d^6^‐DMSO, 500 MHz) spectrum of the solution recovered after the photocatalytic hydrocarboxylation reaction between styrene and an aqueous solution of formate sourced from the anolyte containing EG‐^13^C_2_.

To provide further evidence for the insertion of one carbon atom in the alkyl chain of the synthesized propionic acid derivatives from the different carbon sources used in the electrolysis, we performed isotopic labeling experiments with ^13^CO_2_ and EG‐^13^C_2_. ^13^C‐labeled phenylpropanoate was detected by liquid chromatography‐mass spectrometry (LC‐MS) when the reaction was run with electrogenerated ^13^C‐labeled formate from the catholyte or the anolyte (Figure 6a–c). The possibility to use both formate sources is especially relevant, as it demonstrates that carbon from plastic can also be used in the reaction, as opposed to reported hydrocarboxylation protocols exclusively using CO_2_ as the starting substrate.^[^ ^88^, ^89^, ^90^ ^]^ The results were also confirmed by ^1^H NMR (Figures 6d and S21) and ^13^C NMR spectroscopy (Figure 6e). The signals arising from the protons at C_2_ and C_3_ of phenylpropanoic acid were split into a complex multiplet and a doublet of triplets, respectively, because of the ^13^C–^1^H coupling with the isotopically labeled C_1_ (Figure 6d). Differences in chemical shift are ascribed to pH variability. ^13^C labeling was also visible from the appearance of a strong peak at 177.4 ppm in the ^13^C NMR spectrum (Figure 6e). The assignments were also confirmed by 2D NMR spectroscopy (Figure S22).

## Conclusions

We have reported an enzymatic flow electrolyzer device that can simultaneously perform CO_2_ reduction and oxidation of waste‐derived compounds. Our bespoke flow electrolyzer features a zero‐gap membrane electrode assembly with a unique flow path catered for the flow of CO_2_‐saturated catholyte to the immobilized W‐formate dehydrogenase from *N. vulgaris* Hildenborough. This was made possible by developing a cathode compartment housing a 3D matrix of TiO_2_‐modified carbon felt, which has been specifically designed to provide high enzyme loading in an electroactive configuration, paired with high robustness required for use in a flow electrolyzer configuration. Our biohybrid paired electrolyzer achieves dual formate production at a low cell voltage (−1.5 V) with an initial cell FE of ∼200%, a maximum CO_2_ conversion yield of 18%, and a total operation of 122 h. This performance allowed even for the direct wiring to a commercial silicon photovoltaic (*V*_OC_ = ∼1.5 V) for bias‐free electrocatalytic formate production through CO_2_ and waste comproportionation. We also demonstrate the direct valorization of formate by using the unpurified electrolyte solution following paired electrolysis for the photocatalytic C_1_ chain extension reaction of styrene into phenylpropanoic acid. This sequential electrocatalytic–photocatalytic reaction holds promise for the development of integrated systems for domino conversion of waste‐derived carbon sources into fine chemicals driven by renewable energy in the future.

## Conflict of Interests

The authors declare no conflict of interest.

## Supporting information



Supporting Information

## Data Availability

The data that support the findings of this study are available from the University of Cambridge data repository: https://doi.org/10.17863/CAM.121248.
