# Supplementary material for: Enzymatic Flow Electrolyzer for CO2 and Waste Comproportionation to Formate and Its Use in Photocatalytic Alkene Hydrocarboxylation
Source: Angew Chem Int Ed Engl. 2025 Sep 24;64(46):e202515810. doi: 10.1002/anie.202515810 (PMC12603978; doi:10.1002/anie.202515810)
Supplement: Supplementary file 1 — Supporting Information [file ANIE-64-e202515810-s001.pdf]

Supporting Information

Enzymatic Flow Electrolyzer for CO_2 and Waste Comproportionation to Formate and Its Use in Photocatalytic Alkene Hydrocarboxylation

Beverly Q. L. Low,[1†] Santiago Rodríguez-Jiménez,[1†] Andrea Rogolino,[1] Samuel J. Cobb,[1‡] Chen Han,[1] Guilherme Martins,[2] Inês A. C. Pereira,[2] Erwin Reisner[1*]

[1]Yusuf Hamied Department of Chemistry, University of Cambridge, Lensfield Road, Cambridge CB2 1EW, UK.

[2]Instituto de Tecnologia Química e Biológica António Xavier (ITQB NOVA), Universidade NOVA de Lisboa, Av. da República, 2780-157 Oeiras, Portugal.

*Correspondence to: reisner@ch.cam.ac.uk

[†]These authors contributed equally.

[‡]Current address: Department of Chemistry, The University of Manchester, Oxford Road, Manchester M13 9PL, UK.

Experimental Section

Materials

All chemicals were purchased from commercial suppliers and used without further purification unless otherwise mentioned. Deionised water was obtained from a Simplicity UV MilliQ system (18 MΩ cm) and used in all experiments. Buffers were prepared using tris(hydroxymethyl)aminomethane hydrochloride (Tris-HCl; >99.0%, Sigma), sodium hydrogen carbonate ($NaHCO_3$; >99.998% trace metal basis, Puratronic), potassium chloride (KCl; >99.999% trace metal basis, Fisher Scientific), and potassium hydroxide (KOH, analytical reagent grade, Fisher Scientific).

For the preparation of electrodes, carbon felt (CF, 6.35 mm thick, 99.0%, Thermo Scientific), glacial acetic acid (≥99%, Sigma-Aldrich), Ti foil (0.25 mm thick, 99.7% trace metals basis, Aldrich), absolute ethanol (≥99.8%, Sigma-Aldrich), titanium oxide (TiO_2, P25, Thermo Scientific), graphite powder (APS 7–11 micron, 99%, Thermo Scientific), Araldite standard two component epoxy, nickel foam (1.6 mm thick, PI-KEM) and DL-dithiothreitol (DTT, Ultrapure, Thermo Fischer Scientific) were used.

Ethylene glycol (EG, anhydrous, 99.8%, Sigma-Aldrich), D(+)-glucose (≥99.5%, Sigma), glycerol (99.5%, Thermo Scientific), terephthalic acid (TPA, 99+%, Thermo Scientific Chemicals), glycerol-$^{13}C_3$ (99 atom% ^{13}C, Sigma-Aldrich), D-glucose-$^{13}C_6$ (≥99 atom% ^{13}C, Sigma-Aldrich), ethylene glycol-$^{13}C_2$ (99 atom% ^{13}C, Sigma-Aldrich), $^{13}CO_2$ (99 atom%, Merck), isopropanol (IPA, Honeywell), bipolar membrane (Fumasep FBM), Nafion™ 117 Membrane (Sigma-Aldrich), Parafilm M (Sigma-Aldrich), and rubber septa (Subaseal) were used for electrochemistry experiments.

A commercial lemonade polyethylene terephthalate (PET) bottle and apples (British royal gala apples) were purchased from Sainsbury's and used for the real-world waste experiments. Styrene (Sigma Aldrich, 99%), 4-methoxystyrene (Thermo Scientific, 98%), 4-chlorostyrene (Thermo Scientific, 98%), 4-cyanostyrene (Thermo Scientific, 98%), α-methylstyrene (Sigma Aldrich, 99%), 3-phenylpropionic acid (Sigma Aldrich, 99%), 3-(4-methoxyphenyl)-propionic acid (Thermo Scientific, 98%), 3-(4-chlorophenyl)-propionic acid (Fluorochem), 3-(4-cyanophenyl)-propionic acid (Thermo Scientific, 98%), 3-phenylbutyric acid (Sigma Aldrich, 98%), methyl thiosalycilate (Fluorochem), and potassium formate (Sigma Aldrich, 99%) were used for photocatalytic test and as external standards for product quantification.

Preparation of TiO_2|CF electrodes

The CF ($0.5 \times 0.5 \times 0.64$ cm^3) was cleaned by sonication in a mixture of ethanol and water (1:1) for 20 min and then dried in a 120 ˚C oven for 20 min. A TiO_2 suspension of P25 TiO_2 nanoparticles (10 mg ml^{-1}) was prepared with 5 vol.% acetic acid in ethanol and sonicated for

15 min. The suspension (150 μL) was immediately drop-casted onto the CF and the resultant TiO_2|CF was left to dry briefly (5 min) for the solvent to evaporate. The TiO_2|CF was then annealed at 400 ˚C for 1 h, with a ramp rate of 1 ˚C min^{-1} from room temperature. For measurements using the H-cell, the TiO_2|CF was attached to a Ti foil using a graphite epoxy (GE) paste. To create the GE paste, graphite powder was mixed with Araldite Standard two component epoxy in 3:4 mass ratio.[1] The TiO_2|CF substrate was pressed into the un-set paste and left to dry under ambient conditions overnight. The Ti foil with TiO_2|CF was then attached to a metal T-rod using copper tape, which was then insulated with layers of PTFE tape and parafilm. For measurements using the flow electrolyzer, the TiO_2|CF was used directly.

Preparation of FDH|TiO_2|CF electrode

Nitratidesulfovibrio vulgaris Hildenborough FDH was expressed and purified as previously reported.[2] A solution of DTT (80 mM) was made up in TRIS-HCl buffer (20 mM, pH 7.5). FDH (100 pmol) was incubated with DTT solution (8.4 μL) under inert conditions for 20 min to activate the enzymes. The FDH-DTT solution was then drop-casted onto the TiO_2|CF electrodes and incubated for 2–3 min. To prepare electrodes with different FDH loading, the required amount of FDH was incubated with a proportional amount of DTT solution.

Preparation Ni foam electrode

As-purchased Ni foam (1 × 0.5 cm^2) was pressed into a paper-thin sheet (~0.3 mm) using a vice and then attached to a metal T-rod using copper tape exposing a surface area of 0.5 × 0.5 cm^2. The copper tape was then insulated with layers of PTFE tape and parafilm.

Material characterization

SEM and EDX images were collected using a TESCAN MIRA3 FEG-SEM instrument with an Oxford Instruments Aztec Energy X-maxN 80 system. *In situ* ATR-IR experiments were conducted using a customized spectroelectrochemical cell. Typically, 1 mg of the Ni catalyst was dispersed in a mixture of 1 mL of IPA and 50 μL of 5 wt% Nafion (NR50) under ultrasonication for 30 min to produce an ink. The prepared ink was then painted onto an Au layer, which was sputter-coated onto a Si prism serving as the WE. An Ag/AgCl (saturated NaCl) electrode and a Pt mesh were used as the RE and CE, respectively. The electrolytes used were a solution of 0.1 M KOH with 0.1 vol.% EG or the pretreated PET solution (10 times diluted). All spectroscopic measurements were collected at a resolution of 4 cm^{-1} with at least 128 co-added scans using a PerkinElmer Spectrum 3 FT-IR spectrometer equipped with a liquid nitrogen-cooled MCT detector and a VeeMax III accessory. Electrochemical measurements were performed using an Ivium Compact potentiostat/galvanostat. NMR

spectra for the identification of ^{13}C-labelled phenylpropanoic acid were run on Bruker AVIII HD with DCH cryoprobe (500 MHz) in d^6-DMSO. High-resolution Heteronuclear Single Quantum Coherence (HSQC) spectra were acquired with Water Suppression Enhanced by T1 effects (WET) to suppress the signal from ^{13}C-DMSO.

Pretreatment of real-world wastes

Using an alkaline pretreatment method previously reported,[3] the real-world lemonade bottle made of PET plastic was first cut into small pieces, dipped in liquid nitrogen and pulverized in a grinder. The ground PET plastic pieces were then soaked in 1 M KOH (50 mg mL^{-1}) at 80 ˚C for 72 h under stirring. Similarly, the apples were peeled, cut into small pieces and soaked in water (100 mg mL^{-1}) at 80 ˚C for 24 h, stirring at 500 rpm in air.[4] The pretreated PET and apple solutions were then kept unperturbed at room temperature to cool and allow the unreacted cloudy mass of PET or apple settle down. For the PET solution, the supernatant was directly used as anolyte while the apple solution was added to 1 M aqueous KOH prior to measurements.

H-cell electrochemical characterization

CO_2RR was performed with the FDH|TiO_2|CF cathode in a three-electrode configuration, with a Ag/AgCl (saturated NaCl) RE and Pt wire CE, separated by a Nafion™ membrane. Both compartments of the electrochemical cell were filled with $NaHCO_3$ buffer electrolyte solution consisting of $NaHCO_3$ (100 mM) and KCl (50 mM). The working compartment was purged with CO_2 for 20 min, resulting in a pH of 6.7. Isotopic labelling experiment was conducted using $NaH^{13}CO_3$ (100 mM) and KCl (50 mM) and the working compartment was purged with N_2 for 10 min and evacuated prior to introduction of $^{13}CO_2$ gas.

Anodic substrate oxidation was performed in the same configuration over a Ni foam anode under stirring with a Ag/AgCl (saturated NaCl) RE and Pt wire CE, separated by a Nafion™ membrane. The working compartment was filled with 1 M KOH (pH 14) containing 1 vol.% EG (180 mM), glycerol (140 mM) or 78 mg of glucose (87 mM), while the counter compartment was filled with 1 M KOH. Isotopic labelling experiments were conducted with ^{13}C-labelled substrates (10 mM) in 1 M KOH. CV experiments were performed at a scan rate of 10 mV s^{-1} and controlled potential electrolysis (CPE) experiments were performed for a duration of 1 h using a Biologic VMP potentiostat. The current was reported as a function of the geometrical area of the electrode. All potentials have been quoted vs. RHE using the conversion $E_{RHE} = E_{Ag/AgCl} + 0.197 + (0.059 \times pH)$ V (25 °C).

Product quantification

To quantify the amount of H_2 during the CO_2RR, 50 μL of gas was collected from the headspace and measured using an Agilent 7890A gas chromatograph (GC) equipped with a thermal conductivity detector. Aqueous products were quantified by ^{1}H NMR spectroscopy (Bruker Neo Prodigy 400 MHz) in D_2O with water suppression using maleic acid or 3-(trimethylsilyl)propionic-2,2,3,3,-d_4 acid (TSP) as internal standard. Quantification was done using the MestReNova software where automatic phase and baseline correction were applied. The faradaic efficiency (FE) was calculated using the following equation

$$\mathrm{FE}\ (\%) = \frac{\text{mol of product} \times n \times F}{\text{total charge passed}} \times 100\%$$

where *n* is the electron transfer number per mole of product and *F* is the Faraday's constant (96485 C mol^{-1}). n = 2 for H^+ to H_2, 2 for CO_2 to formate, 3 for EG to formate, 2.67 for glycerol to formate, 4 for glycerol to glycerate, 3.33 for glycerol to glycolate and 2 for glucose to formate.[5]

Trace amounts of formate present in the pretreated PET solution was subtracted during the calculation of FE. For substrates such as glucose and pretreated apple solution, the amount of formate produced *via* hydrolysis (without any applied potential) was subtracted to accurately quantify the amount of formate produced solely from electrochemical oxidation.

Styrene derivatives and corresponding propionic acid derivatives were quantified by HPLC with external standards (Figure S23) on a Waters Breeze system equipped with a diode array UV–vis (λ = 254 nm) detector using a Phenomenex Luna Omega C18 column at 40°C. Samples were analyzed in the isocratic flow mode (flow rate 0.5 mL min^{-1}, H_2O/MeCN 1:1).

Molecular mass was determined by LC-MS on a Thermo Fisher Q Exactive in negative polarity mode with 3.00 kV spray voltage and 320 ˚C capillary temperature.

Paired electrolysis

Continuous-flow electrolysis of FDH|TiO_2|CF and Ni foam was carried out in a homemade electrolyzer in two-electrode configuration. The anode compartment of the homemade electrolyzer consists of a stainless-steel anode flow plate and backplate with the PTFE gasket sandwiched in between. The Ni foam anode is placed between the anode flow plate and a BPM which is held in place by a Viton gasket. The cathode flow plate was 3D printed (Formlabs Form 3B+) with a low force stereolithography method (layer height of 50 μm) using rigid glass fiber reinforced resin (Rigid 10k) for reduced gas permeability. The FDH|TiO_2|CF cathode is fitted into the square hole at the center of the cathode flow plate and a piece of graphite back contact is placed between the flow plate and backplate, ensuring contact between the spring pin in the backplate and the carbon felt. M4 × 45 mm bolts (8) were used to clamp the anode and cathode compartments together. M4 × 15 mm hex head set screws (6) were used to

secure the cathode backplate to the flow plate. The electrolyte solutions were flown through the ¼ inch UNF (¼-28) threaded inlet and outlet ports on the cathode and anode backplates at a flow rate of 0.5 cm^3 min^{-1} using a peristaltic pump (Ismatec) and tubing (Masterflex) that have low gas permeability. The catholyte, $NaHCO_3$ (100 mM) and KCl (50 mM), was continuously purged with humidified CO_2 gas and the anolyte consisted of 1 vol.% of substrate in 1 M KOH or as-prepared pretreated PET or apple solutions. The electrochemical measurements were performed using a Ivium Compact potentiostat in a N_2-filled glovebox at ambient temperature (25 ˚C) and paired electrolysis was conducted at a full-cell voltage of −2.5 V or −1.5 V. For the long-term flow electrolysis at a full-cell voltage of −1.5 V, the catholyte was purged with CO_2 for 20 min in the beginning and at respective time intervals. PV-powered electrolysis experiments were conducted using 3 Si cells in series (1 × 4.5 cm^2) illuminated by an LED light source (0.1 sun, 10 mW cm^{-2}).

Synthesis of 2,4,5,6-tetrakis(diphenylamino)isophthalonitrile (4DPAIPN)

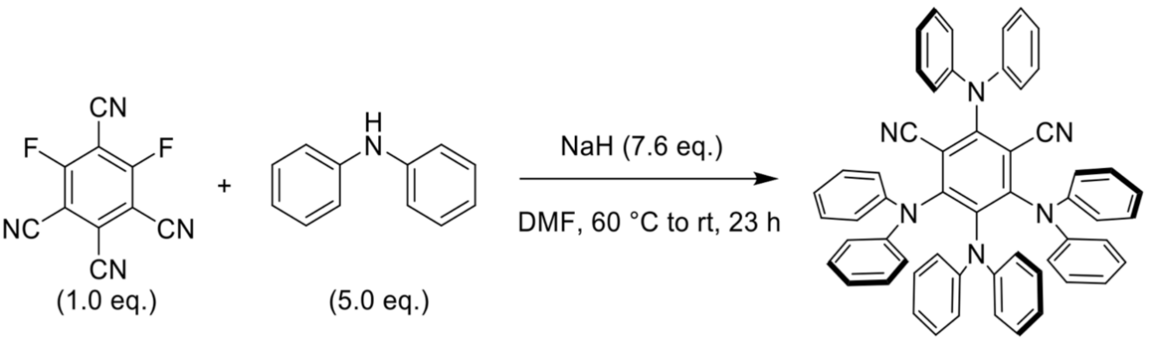

The synthesis was adapted from a reported procedure.[6] In an anhydrous glove box, 1.42 g of 60% oil dispersion in paraffin of sodium hydride (Fluorochem, 35.5 mmol, 7.6 equiv.) were added to 70 mL of anhydrous DMF in a 250 mL round-bottomed flask. Then, a solution of 4.0 g (23.6 mmol, 5.0 equiv.) of diphenylamine (Sigma Aldrich, ≥99%) in 12 mL of anhydrous DMF was added dropwise. The flask was sealed with a rubber septum, taken out of the glove box and heated to 60 °C for 30 min, venting occasionally to prevent pressure build-up. After this time, 12 mL of a solution of 0.95 g (4.7 mmol, 1.0 equiv.) of tetrafluoroisophthalonitrile (Fluorochem) were added dropwise with a syringe through the rubber septum. The solution was allowed to stir at 40 °C for 10 h, then stirred for additional 12 h at room temperature. After this time, the reaction was quenched with 10 mL of deionized water, and the mixture was poured into 100 mL of deionized water. The mixture was filtered, washed with water. The solid was redissolved in dichloromethane, the solvent was evaporated under reduced pressure, and the product was recrystallized in dichloromethane using hexane as anti-solvent. The

product was filtered and washed with hexane. The residual solvent was removed under vacuum at room temperature, yielding an orange powder (2.57 g, 67%).

Photocatalytic alkene hydrocarboxylation

In a typical test for reaction optimization, styrene (5 μmol), methyl thiosalicylate (0.10 μmol), 4DPAIPN (0.005 μmol), and potassium formate (5.5 μmol) were added to 0.5 mL of DMSO/H_2O with different solvent proportions in a 11 mL reaction vial. The solution was purged with N_2 for 30 min. The vial was irradiated under blue light (λ = 450 nm, 80±13 mW) at 25 °C for 4 h. Variations to the standard conditions described here are reported in the captions or footnotes of relevant tables.

In a typical test with electrogenerated formate, styrene (0.5 μmol), methyl thiosalicylate (0.05 μmol), 4DPAIPN (0.005 μmol) were added to a mixture of 900 μL DMSO and 100 μL of aqueous electrolyte solution containing 2–4 equiv. (1.0–2.0 μmol) of formate in a 11 mL reaction vial. The aqueous solution from the electrolyzer was occasionally concentrated by solvent evaporation when unable to generate sufficient formate at sufficient molarity (*i.e.,* < 10 mM). Anolyte samples were previously neutralized to pH 6–7 with 1 M HCl. Catholyte samples were added with no further modifications. The solution was purged with N_2 for 30 min. The vial was irradiated under blue light (λ = 450 nm, 80±13 mW) at 25 °C for 16 h.

Data analysis

All electrochemical experiments were conducted in triplicates. The results are represented with the mean ($\bar{x}$) and the standard deviation ($\sigma_{\bar{x}}$) expressed as $\bar{x}\pm\sigma_{\bar{x}}$, calculated using equations (1) and (2) below.

$$\bar{x} = \frac{1}{n}\sum_{i=1}^{n} x_i \qquad \text{(Eq. 1)}$$

$$\sigma_{\bar{x}} = \sqrt{\frac{1}{(n-1)}\sum_{i=1}^{n}(x_i - \bar{x})^2} \qquad \text{(Eq. 2)}$$

where n is the number of measurements and x_i the individually determined value.

Notes on CO_2 conversion calculation

All calculations were based on a temperature of 298 K (25 ˚C) and 1 atm pressure. When CO_2 is purged into the catholyte (100 mM $NaHCO_3$ with 50 mM KCl), an equilibrium will be established:

$$CO_{2\,(g)} \rightleftharpoons CO_{2\,(aq)} + H_2O_{\,(l)} \rightleftharpoons HCO_3^-{}_{(aq)} + H^+{}_{(aq)} \rightleftharpoons CO_3^{2-}{}_{(aq)} + 2\,H^+{}_{(aq)}$$

Assuming CO_2 behaves as an ideal gas, the solubility of CO_2 will follow Henry's Law,

$$k = \frac{[CO_2]_{aq}}{P_{CO_2}}$$

where k is Henry's law constant (3.1×10^{-2} M atm^{-1} for CO_2 in water at 25 ˚C),[7] $[CO_2]_{aq}$ is the concentration of dissolved CO_2 and P_{CO_2} is the partial pressure of CO_2 (1 atm).

As shown in previous work,[8] the 'salting out' effect of ionic species, (HCO_3^-), (CO_3^{2-}), (K^+) and (Cl^-), in the catholyte will result in a decrease in CO_2 concentration at a given pH. Furthermore, the bulk pH measured at the start and end (~122 h) of the experiment increased from pH 6.7 to 7.8, indicating the equilibrium shifts away from CO_2 and towards HCO_3^- as the experiment progresses. While we acknowledge that the actual concentration of CO_2 in the catholyte will be lower than the theoretical concentration at saturation (31 mM), the CO_2 concentration was assumed to be 31 mM for simplicity. The amount of CO_2 introduced during each round of purging was then calculated using the catholyte volume at respective time intervals. As such, the calculated CO_2 conversion values are an underestimate of the true values.

Table S1. Comparison of performance metrics across CO_2 electrolyzers for paired formate production. (F) = foam, HOS = surface sulfurized. The cell FE refers to the combined FE towards formate of both the cathodic and anodic reactions.

**CO_2 electrolysers for paired formate production**	**Oxidation**	**Cell voltage (V)**	**Cell FE (%)**	**Stability (h)**	**Current density (mA cm^{-2})**	**Ref.**
Cu(F)|Pb-SnO||Cu(F)|CuO|$Ni(OH)_2$	EGOR	1.85	89.6	24	20	[9]
BiOBr||Ni_xB|Ni(F)	GlyOR	8.8	141	0.25	200	[5]
BiOI|$Ni_{0.33}Co_{0.67}(OH)_2$|HOS|Ni(F)	GlyOR	1.8	~180	36	10	[10]
SnO_2||$NiCo_2O_4$	PET-OR	1.9	155	2	20	[11]
$Bi_2O_2CO_3$|rGO||CuCoO|rGO	PET-OR	1.9	152	1	10	[12]
Bi|Bi_2O_3||$Ni(OH)_2$	PET-OR	2.7	181	4.17	100	[13]
BiOI-C||$NiCo_2O_4$	PET-OR	2.7	175	140	250	[14]
In_2O_3|CP||SnO_2|Ni(F)	PET-OR	2.9	182	30	180	[15]
Bi(1.5 S)||NiOOH/$Ni_3Bi_2S_2$	PET-OR	3	>180	45	15.5	[16]
$Bi_2O_2CO_3$||Mn|CoOOH	PET-OR	2.2	184	100	150	[17]
FDH|TiO_2|CF||Ni(F)	EGOR	2.5[1]	~200	1	5	This work
FDH|TiO_2|CF||Ni(F)	PET-OR	2.5[1]	197	1	~4	This work
FDH|TiO_2|CF||Ni(F)	GlyOR	2.5[1]	180	1	4.5	This work
FDH|TiO_2|CF||Ni(F)	EGOR	1.5[1]	166	122	1	This work

[1]Cathode used as working electrode, cell voltage given as absolute values.

Table S2. Comparison of performance metrics across systems FDH-immobilized electrodes for CO_2 reduction. PVK = perovskite, GDE = gas diffusion electrode, PANi = polyaniline, IO = inverse opal, OPV = organic photovoltaic.

FDH-immobilized systems for CO_2 reduction	Overall Formate FE (%)	Stability (h)	Rate of formate production (μmol h^{-1} cm^{-2})	Ref
$BiVO_4$||PVK|IO-TiO_2|FDH	83	10	7	[1]
FDH|PANi hydrogel	92.7	12	1.42	[18]
FDH immobilized on viologen-modified redox polymer GDE	NA	48	0.056	[19]
FDH|IO-TiO_2|OPV||Zr:α-Fe_2O_3|Ni$(OH)_x$	176	10	11	[20]
FDH|TiO_2|C(F)||Ni(F)	166	122	5.93	This work

Table S3. Accumulated amounts of formate produced, and CO_2 introduced at respective time intervals, as well as the calculated CO_2 conversion during long term paired electrolysis of CO_2RR with EGOR, n = 3. The difference in CO_2 amounts arises from differences in catholyte volume due to evaporation losses or spillages. More details in **Notes on CO_2 calculation**.

Time (h)	Total amount of formate produced (μmol)	Total amount of CO_2 introduced (μmol)	CO_2 conversion (%)
4.7	25.7±3.5	310±0	8.3±1.2
26.7	90.5±11.8	572±21.1	15.8±2.2
50.7	140±9.2	806±28.2	17.7±1.5
74.7	157±10.9	1008±26	16.3±1.5
98.7	167±23.3	1192±27.9	14.8±1.5
122.1	164.6±16.9	1361±34.2	13.3±1.5

Table S4. Optimization of photocatalytic styrene hydrocarboxylation with potassium formate. Other conditions: Styrene (5 μmol), $KHCO_2$ (5.5 μmol, 1.1 equiv.), 4DPAIPN (0.1 mol.%), methyl thiosalycilate (2 mol.%), 0.5 mL solvent (DMSO/H_2O 9:1), 1 atm N_2, 25 °C, 4 h.

Entry	H_2O (Vol.%)	Irradiation[1]	Buffer	Phenylpropanoic acid yield (%)[2]	Styrene conversion (%)[2]
1	1	450 nm	None	88±1	78±8
2	2	450 nm	None	73±4	100
3	5	450 nm	None	62± 2	100
4	10	450 nm	None	63±11	100
5	15	450 nm	None	0	53±2
6	20	450 nm	None	0	50±2
7	10	450 nm	0.1 M $KHCO_3$[3]	91±2	100
8	10	AM 1.5G	None	50±20	97±4
9	10	AM 1.5G	0.1 M $KHCO_3$[3]	47±9	94±8

[1]450 nm LED: 80±13 mW, AM 1.5G: 100 mW cm^{-2} (1 Sun).
[2]Error bars are calculated as the standard deviations of duplicates.
[3]The concentration exceeds the solubility of $KHCO_3$ in the DMSO/H_2O mixture.

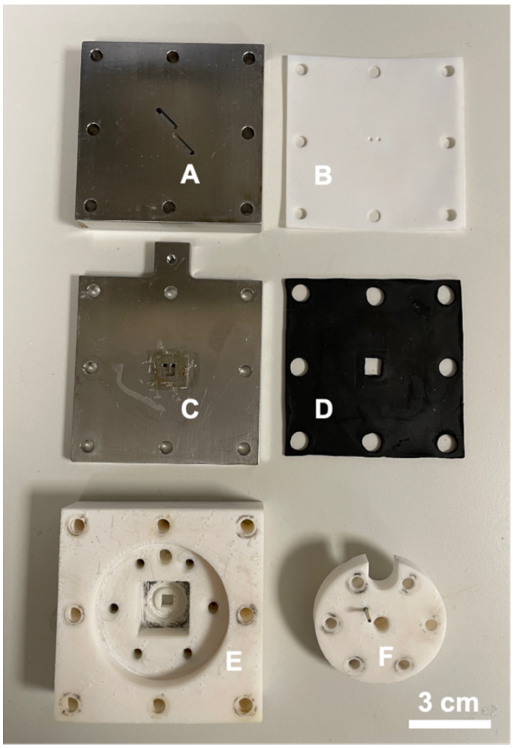

Figure S1. Image of individual components of the flow electrolyzer; A: anode backplate, B: PTFE gasket, C: anode flow plate, D: Viton gasket, E: cathode flow plate, F: cathode back plate. Components A and C were mechanically machined from stainless-steel while components E and F were 3D printed using a layer-by-layer stereolithography method with rigid glass fiber reinforced resin.

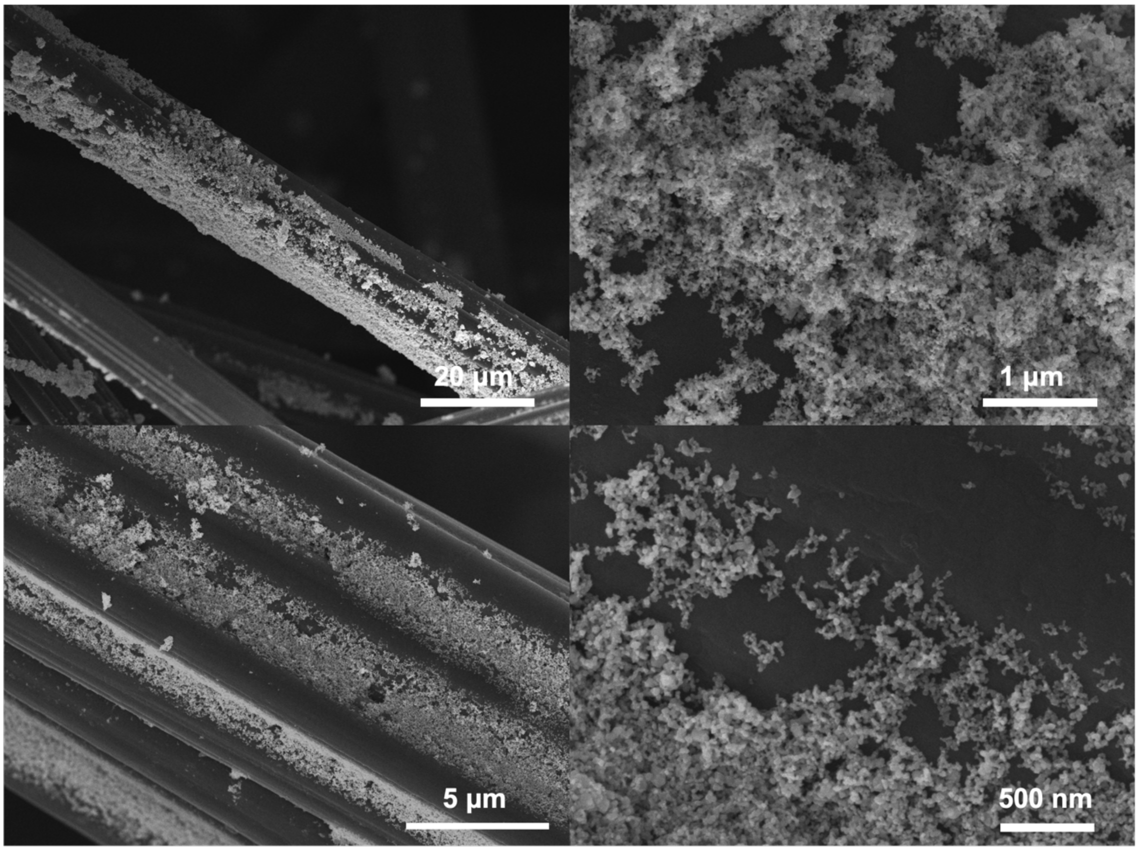

Figure S2. Scanning electron microscopy (SEM) images of TiO_2-coated carbon felt electrodes (TiO_2|CF) with different magnifications (indicated in the figure), depicting the TiO_2 nanoparticles on the carbon fibers.

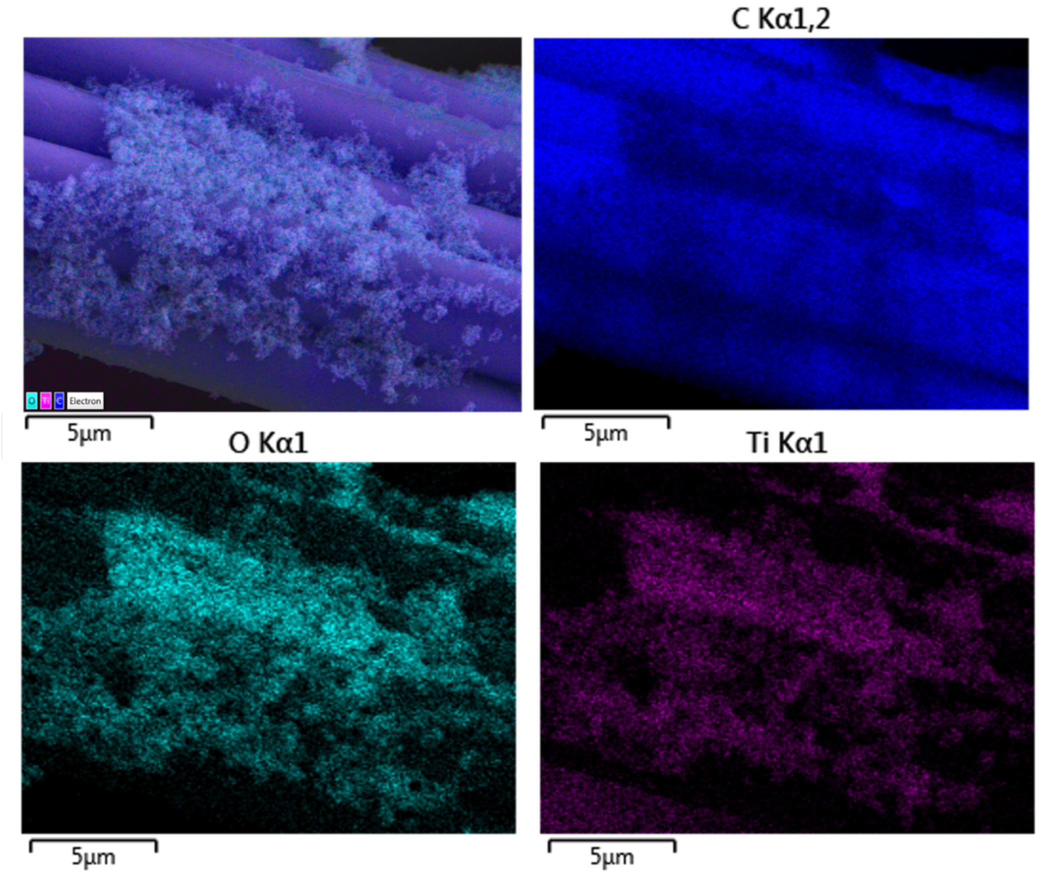

Figure S3. Energy-dispersive X-ray spectroscopy (EDX) of TiO_2|CF electrodes, where agglomerates of mesoporous TiO_2 nanoparticles are visible on the carbon fiber, as confirmed by the presence of O and Ti elements.

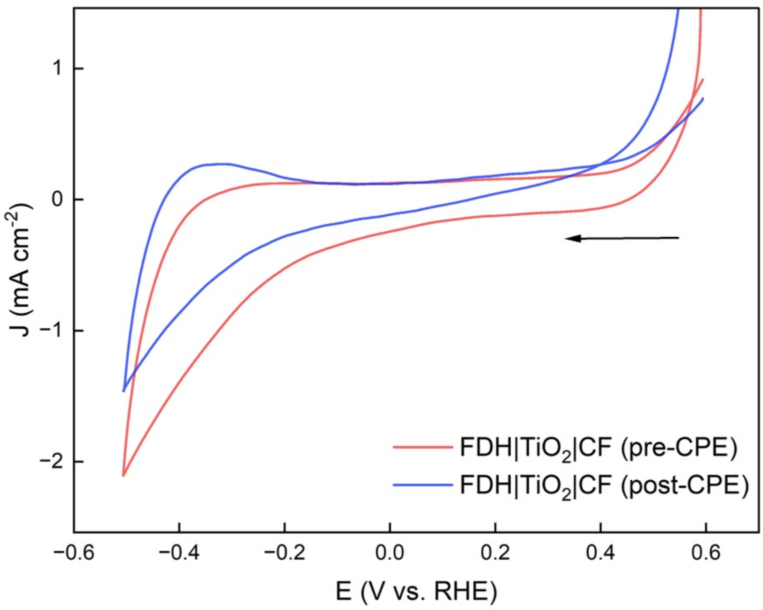

Figure S4. Post characterization of FDH|TiO$_2$|CF. CV curve before (pre) and after (post) 1h of CPE at −0.5 V vs. RHE (3-electrode configuration), showing a reduction in current density as well as the appearance of anodic peak corresponding to the discharging of the conduction band of TiO$_2$.[21] This is congruent with the observed hydrogen evolution reaction and decrease in FE towards formate at more negative applied potentials. Conditions: 100 pmol FDH loading, 100 mM NaHCO$_3$ with 50 mM KCl (5 mL) was used as the electrolyte and purged with CO$_2$ for 20 min prior to measurement (pH 6.7, 25 ˚C).

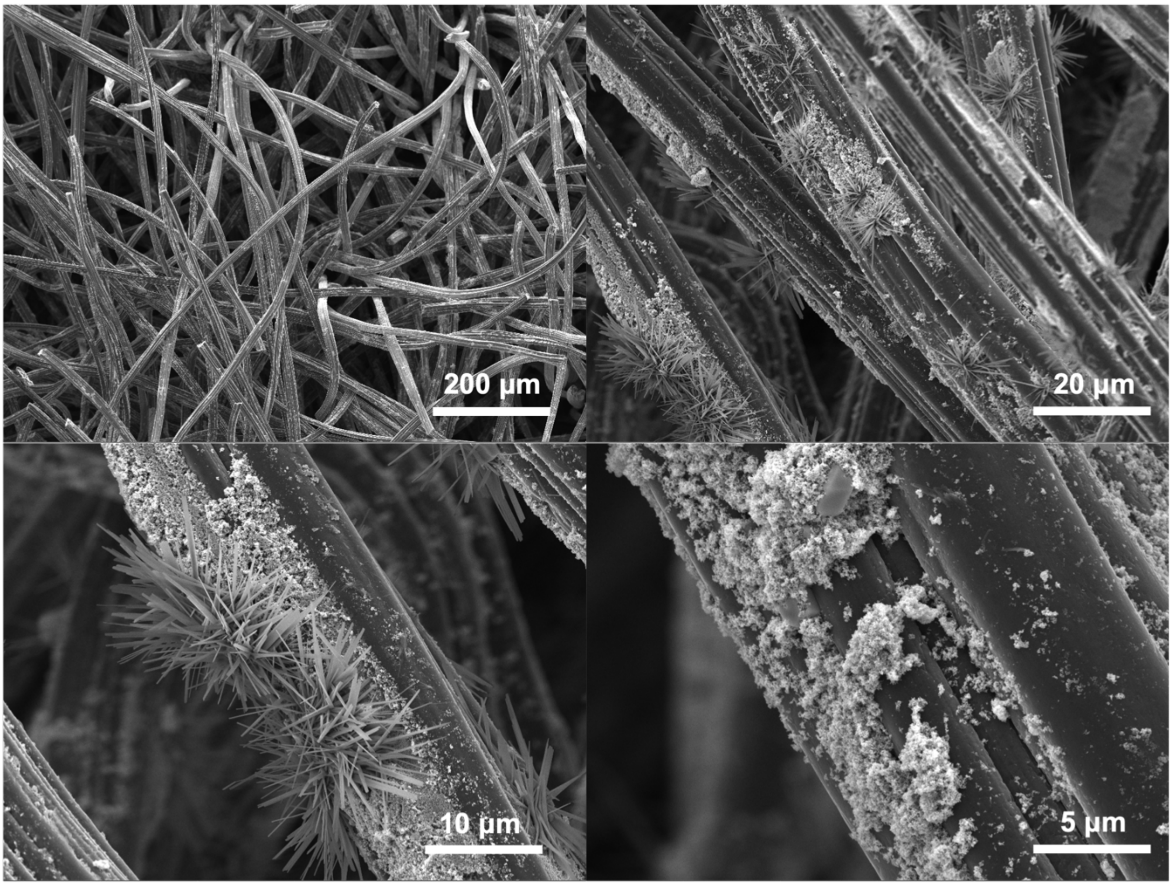

Figure S5. Post characterization of FDH|TiO_2|CF. SEM images of FDH|TiO_2|CF electrodes after CPE at −0.5 V vs. RHE (3-electrode configuration), with different magnifications (indicated in the figure), showing similar morphology of TiO_2 nanoparticles compared to the pristine TiO_2|CF electrode. Clusters of spike-like structures are observed and can be attributed to the deposition of salts from the buffer ($NaHCO_3$ with KCl).

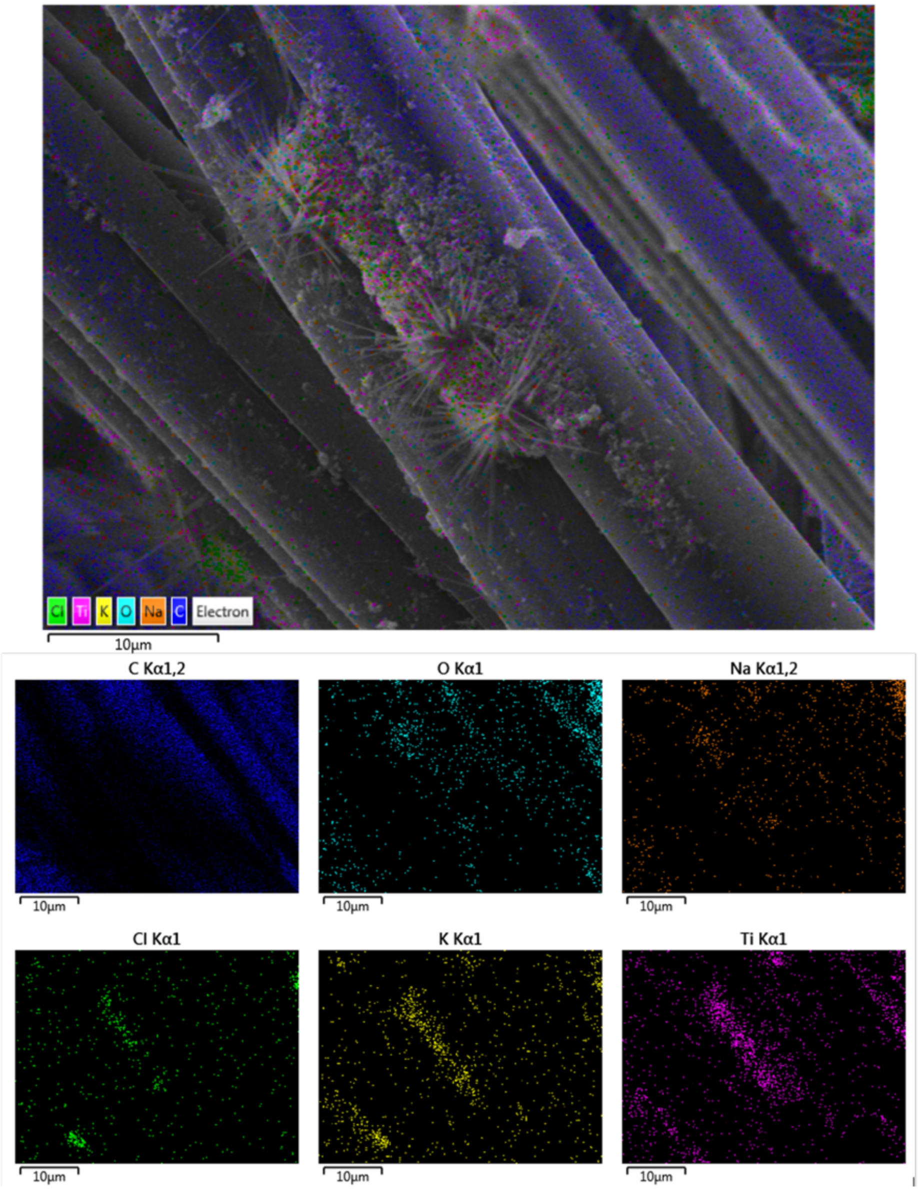

Figure S6. Post characterization of FDH|TiO_2|CF. EDX of FDH|TiO_2|CF electrodes after CPE at −0.5 V vs. RHE (3-electrode configuration) shows the presence of Na, K and Cl and confirms the spike-like structures observed are due to the salts in the buffer (KCl).

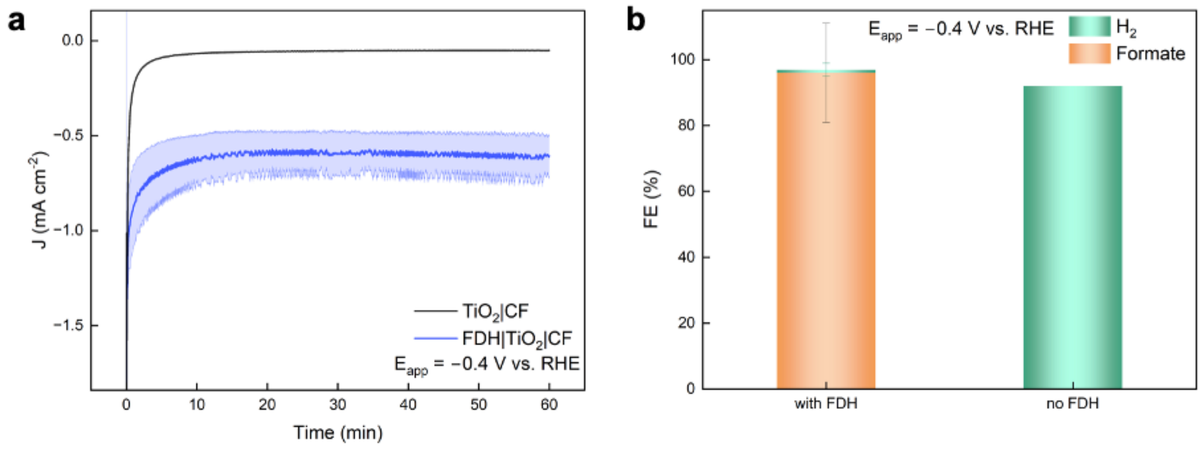

Figure S7. (a) Controlled potential electrolysis (CPE) of FDH|TiO_2|CF compared with bare TiO_2|CF at applied potential of −0.4 V vs. RHE, and (b) the corresponding product distribution and FE. In the absence of FDH, no amount of formate was detected and hydrogen evolution on TiO_2 takes places instead. Conditions: 100 pmol FDH loading, 100 mM $NaHCO_3$ with 50 mM KCl (5 mL) was used as the electrolyte and purged with CO_2 for 20 min prior to measurement (pH 6.7, 25 ˚C).

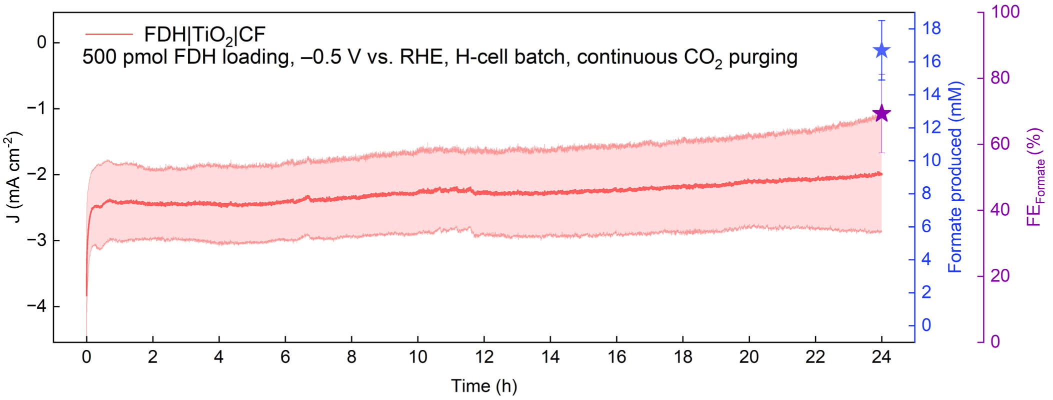

Figure S8. Preliminary CPE of FDH|TiO_2|CF to survey the long-term stability and selectivity at −0.5 V vs. RHE in batch mode. Conditions: 500 pmol FDH loading, 100 mM $NaHCO_3$ with 50 mM KCl (10 mL), continuous CO_2 purging.

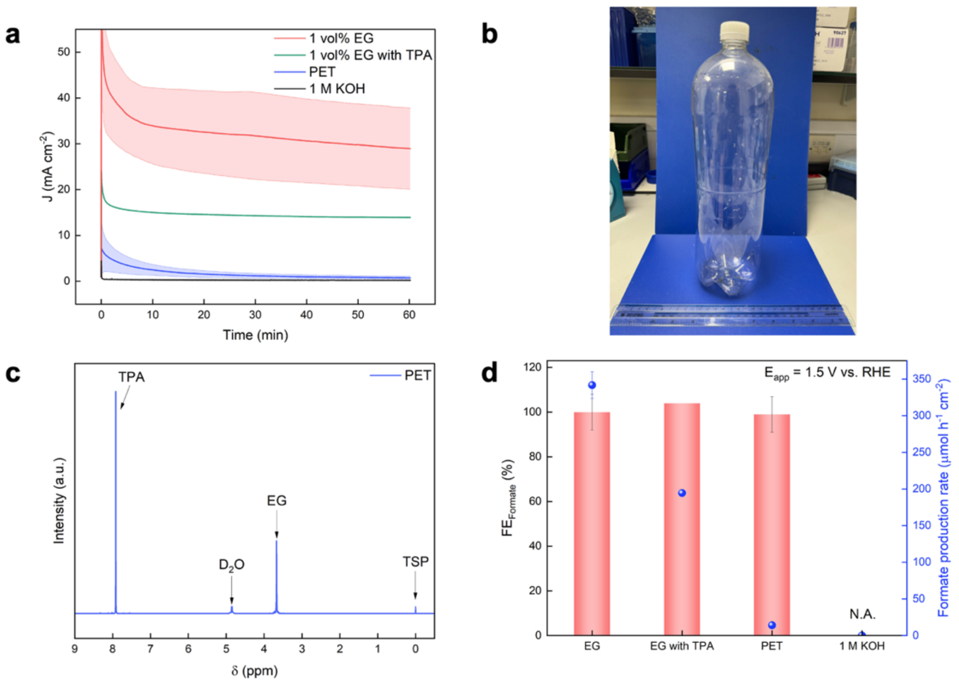

Figure S9. (a) CPE over Ni foam in the presence of different substrates and no substrate (1 M KOH) at an applied potential of 1.5 V vs RHE. (b) Image of polyethylene terephthalate (PET) bottle used as real-world waste to obtain EG via alkaline pretreatment, and (c) the ^{1}H NMR spectrum of different constituents in the pretreated PET solution measured using 3-(trimethylsilyl)propionic-2,2,3,3,-d_4 acid (TSP) as internal standard (0.04 wt.%). (d) FE towards formate and rate of formate production during 1 h of CPE. The control with added terephthalate (TPA, 0.193 M) mimics the composition of the pretreated PET solution and illustrates how the presence of TPA lowers current density yet has no effect on the ~100% FE towards formate. No formate was produced in the control without any substrate, confirming EG as the source of formate. Conditions: 1.5 V vs. RHE applied potential, 1 vol.% of EG in 1 M KOH (pH 14, 5mL) or as-prepared PET solution were used as electrolyte, ambient temperature (25 ˚C).

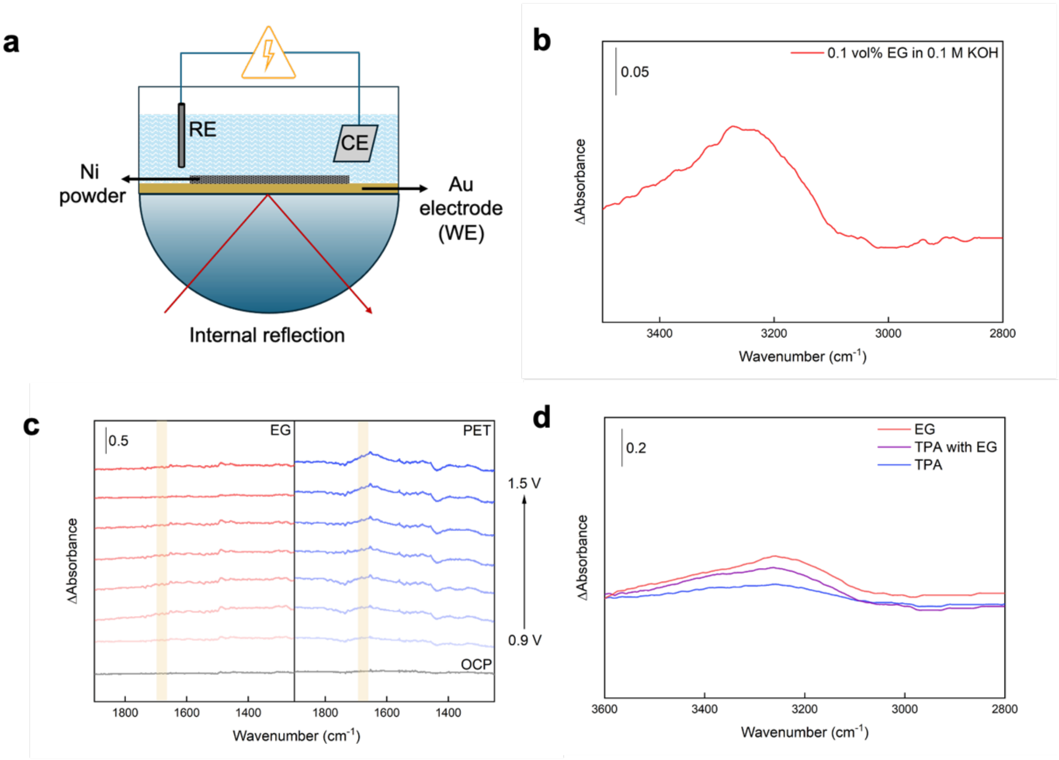

Figure S10. (a) Schematic diagram of the setup used for *in situ* IR measurements. (b) IR spectra of 0.1 M KOH solution with EG from which the increase in peak intensity at 3255 cm^{-1} suggests the adsorption of EG on the Ni powder. ΔAbsorbance was calculated by subtracting the background scan measured with 0.1 M KOH. While the broad peak from 3200 to 3400 cm^{-1} is attributed to overlapping contributions of surface-bound OH groups and EG, a differential analysis of the spectrum in 0.1 M KOH from that in 0.1 M KOH + EG yields a distinct positive band around ≈3255 cm^{-1}, indicating additional contribution from EG adsorption beyond that of water. (c) *In situ* IR spectra measured at successive applied potentials using electrolyte containing 0.1 vol% EG in 0.1 M KOH (red) and 10 times diluted PET solution (blue). ΔAbsorbance was calculated by subtracting the background scan measured without applied potential (OCP). Ni powder, Ag/AgCl and Pt wire were used as the working, reference and counter electrodes respectively. The peak at 1680 cm^{-1} corresponds to the C=O stretching vibration of the –COOH group of TPA.[22] (d) IR spectra of a solution of TPA, EG or both in 0.1 M KOH on Ni powder, where the presence of TPA appears to lower the peak owing to EG adsorption. ΔAbsorbance was calculated by subtracting the background scan measured with deionized water.

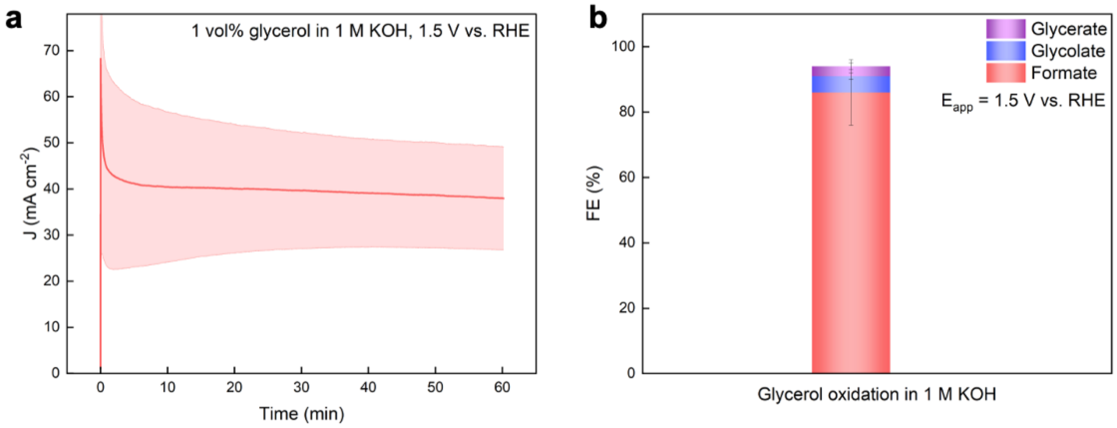

Figure S11. (a) CPE of Ni foam in 1 vol% glycerol in 1 M KOH (pH 14, 5 mL) at applied potential of 1.5 V vs. RHE (ambient temperature, 25 ˚C) and correspondingly, b) the product distribution and FE towards each product for 1 h of CPE. The experiment was performed in triplicates where the mean is shown with errors represented as the shaded region or bars.

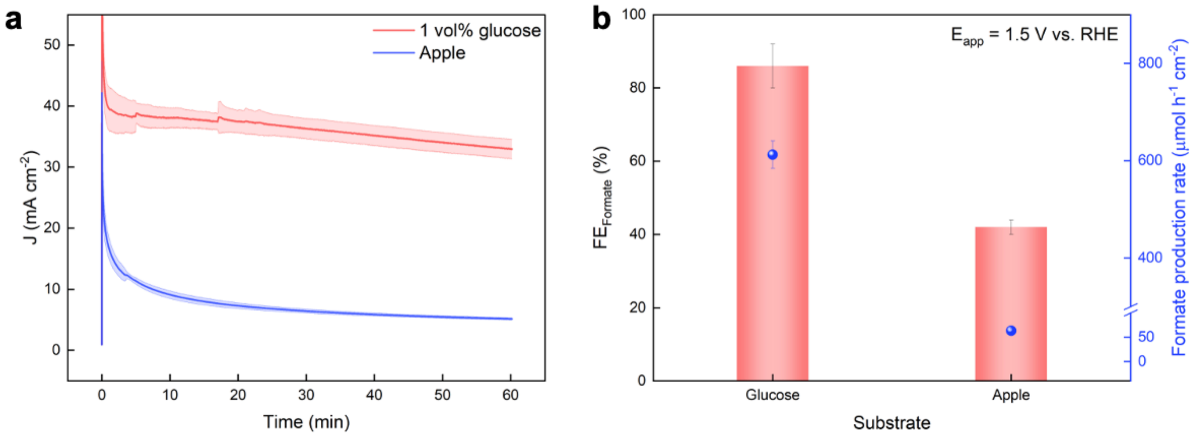

Figure S12. (a) CPE of Ni foam in 1 vol% glucose and pretreated apple solution in 1 M KOH (pH 14, 5 mL) at applied potential of 1.5 V vs. RHE (ambient temperature, 25 ˚C) and correspondingly, (b) the FE towards formate and rate of formate production for 1 h of CPE. The experiment was performed in triplicates where the mean is shown with errors represented as the shaded region or bars.

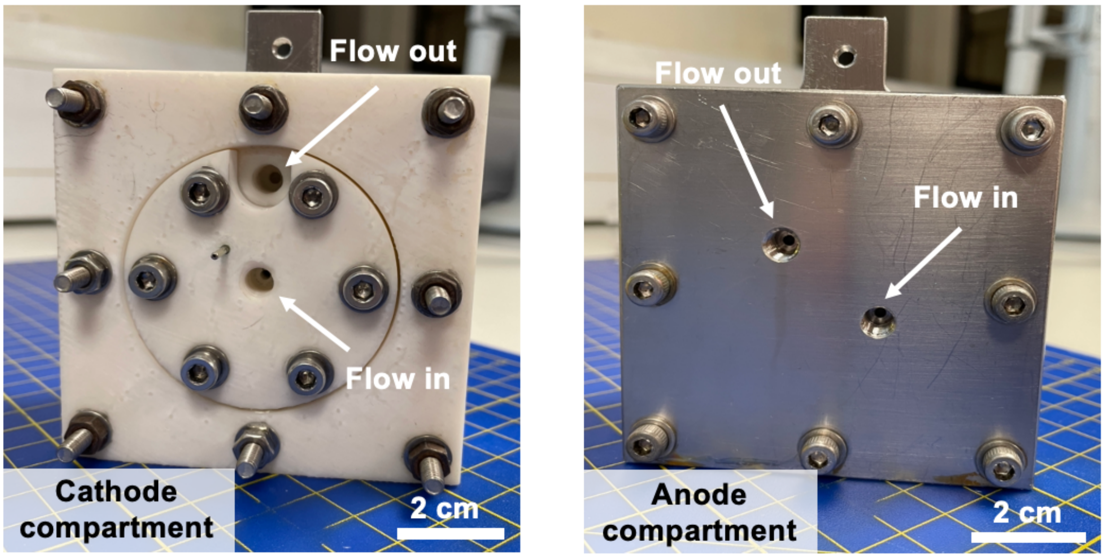

Figure S13. Pictures of the assembled flow electrolyzer with labelled flow inlets and outlets at the respective anode and cathode compartments.

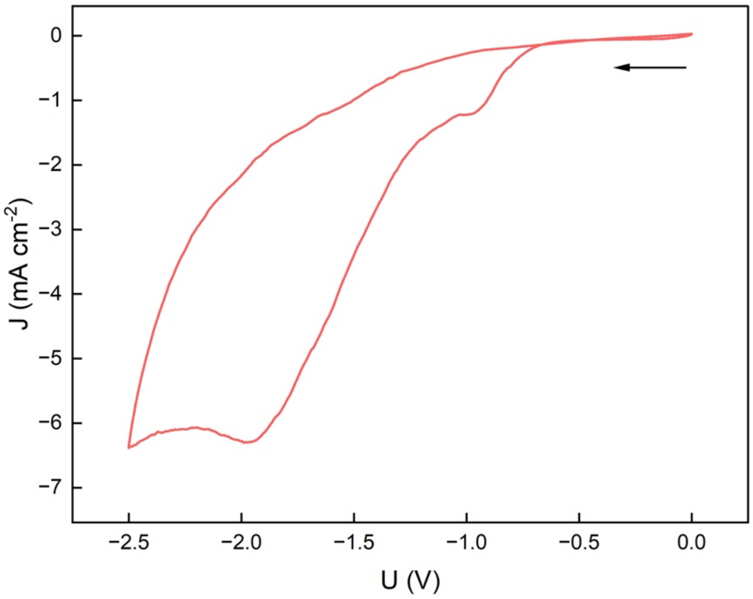

Figure S14. CV of paired electrolysis of CO_2 and EG without FDH. Conditions: bare TiO_2|CF cathode with Ni foam anode, ambient temperature (25˚C), catholyte (100 mM $NaHCO_3$ with 50 mM KCl) and anolyte (1 vol.% EG in 1 M KOH) were constantly flowing through the respective compartments at a rate of 0.5 mL min^{-1} and a BPM was used.

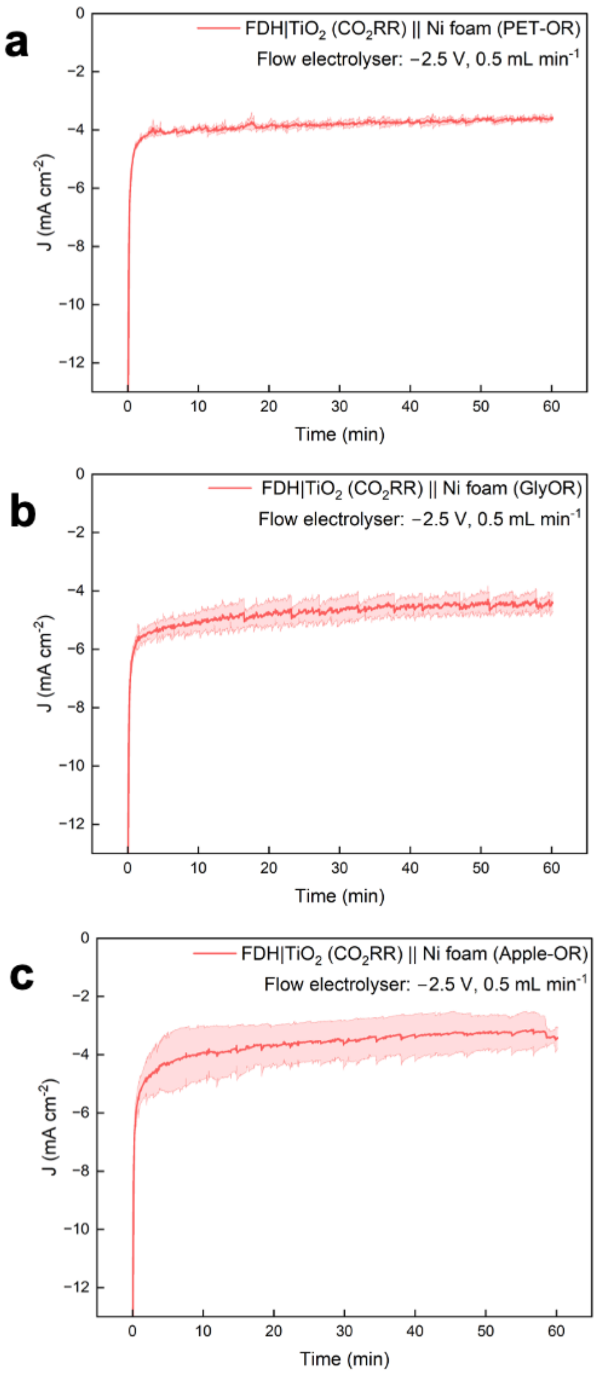

Figure S15. Paired electrolysis at an applied voltage of −2.5 V for CO_2 reduction paired with (a) PET oxidation reaction (PET-OR), (b) glycerol oxidation reaction (GlyOR) and (c) apple solution oxidation reaction (Apple-OR). Conditions: 500 pmol FDH loading, catholyte (100 mM $NaHCO_3$ with 50 mM KCl, 10 mL, continuously purged with CO_2) and anolyte (1 vol% glycerol in 1 M KOH or as-prepared PET and apple solutions, 10 mL) were constantly flowing through the respective compartments at a rate of 0.5 mL min^{-1}. A voltage of −2.5 V was applied, and a bipolar membrane was used.

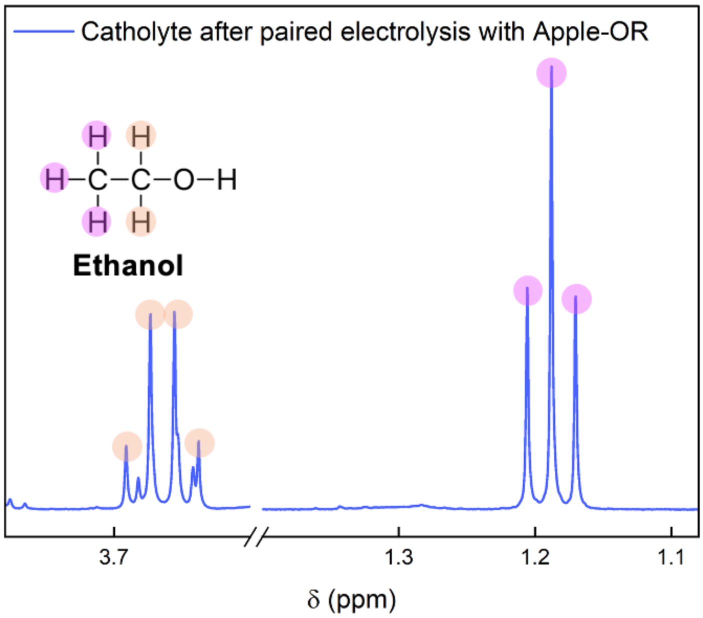

Figure S16. ^{1}H NMR spectrum (D_2O, 400 MHz) of the catholyte after paired electrolysis with Apple-OR. The peaks correspond to the protons of ethanol as indicated in the respective colors. From the integration of the peaks (not shown), the concentration of ethanol is about 2 mM. Conditions of paired electrolysis: 500 pmol FDH loading, ambient temperature (25˚C), catholyte (100 mM $NaHCO_3$ with 50 mM KCl, 10 mL) and anolyte (as-prepared apple solution, 10 mL) were constantly flowing through the respective compartments at a rate of 0.5 mL min^{-1}. A voltage of −2.5 V was applied, and a bipolar membrane was used.

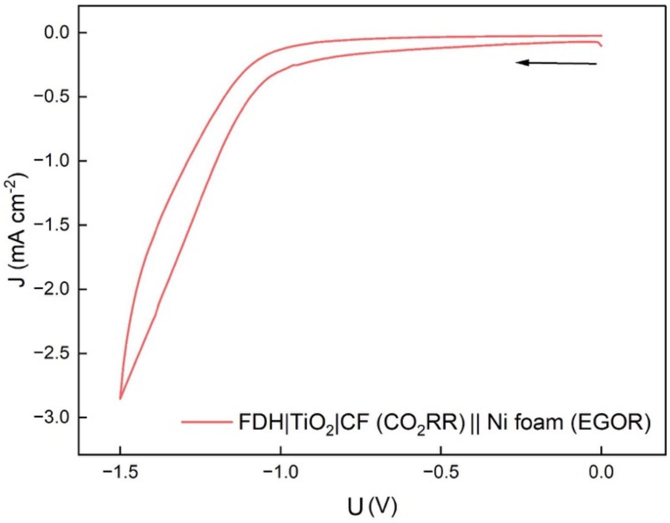

Figure S17. Cyclic voltammetry of flow electrolyzer for CO_2 reduction reaction (CO_2RR) paired with ethylene glycol oxidation reaction (EGOR). Conditions: 500 pmol FDH loading, ambient temperature (25˚C), catholyte (100 mM $NaHCO_3$ with 50 mM KCl, 10 mL) and anolyte (1 vol.% EG in 1 M KOH, 10 mL) were constantly flowing through the respective compartments at a rate of 0.5 mL min^{-1}.

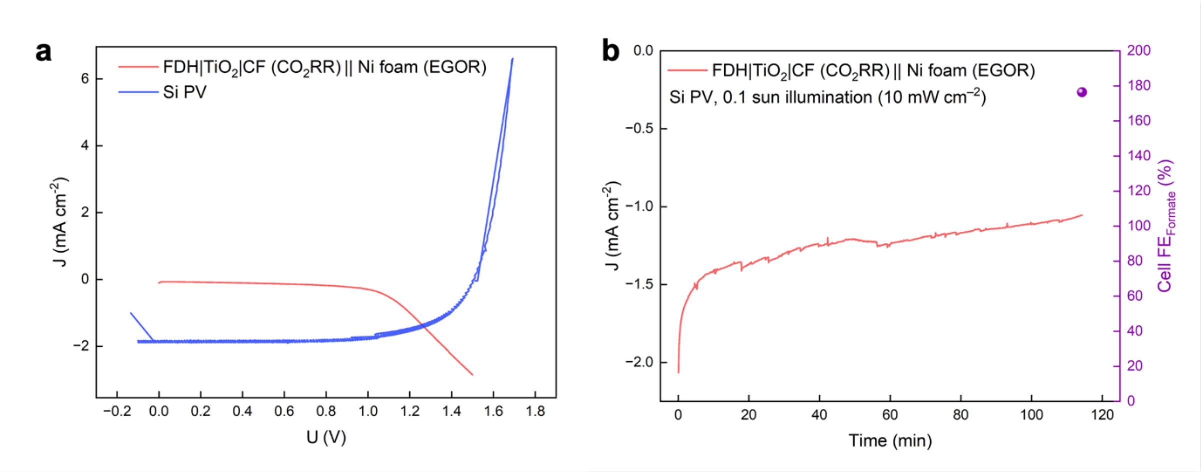

Figure S18. (a) J-V curve showing the intersection between the 2-electrode flow electrolyzer and the Si photovoltaic (PV, 10 mW cm^{-2} white LED irradiation) and (b) photoelectrolysis trace of the PV-powered electrolyzer under bias-free conditions. Conditions: 500 pmol FDH loading, catholyte (100 mM $NaHCO_3$ with 50 mM KCl, 10 mL) and anolyte (1 vol% EG in 1 M KOH, 10 mL) were constantly flowing through the respective compartments at a rate of 0.5 mL min^{-1}. The catholyte was purged with CO_2 for 20 mins prior to the start of electrolysis.

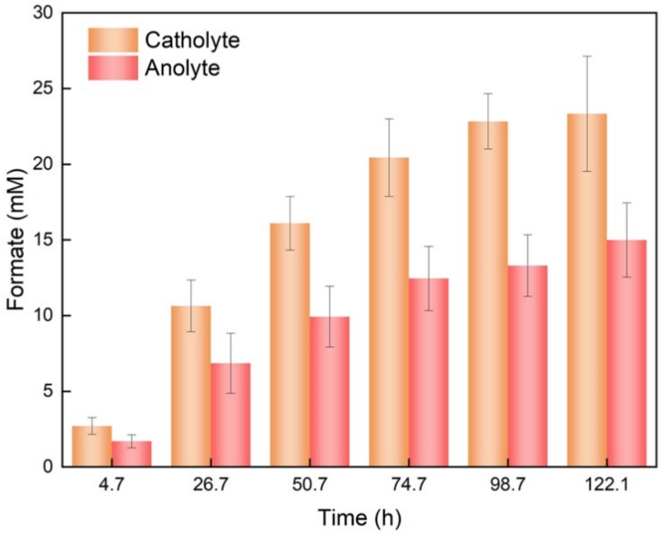

Figure S19. Concentration of formate in catholyte and anolyte measured at different time intervals during the long-term paired electrolysis of CO_2 and EG. Conditions: 500 pmol FDH loading, catholyte (100 mM $NaHCO_3$ with 50 mM KCl, 10 mL) and anolyte (1 vol% EG in 1 M KOH, 10 mL) were constantly flowing through the respective compartments at a rate of 0.5 mL min^{-1}. A voltage of −1.5 was applied and a bipolar membrane was used.

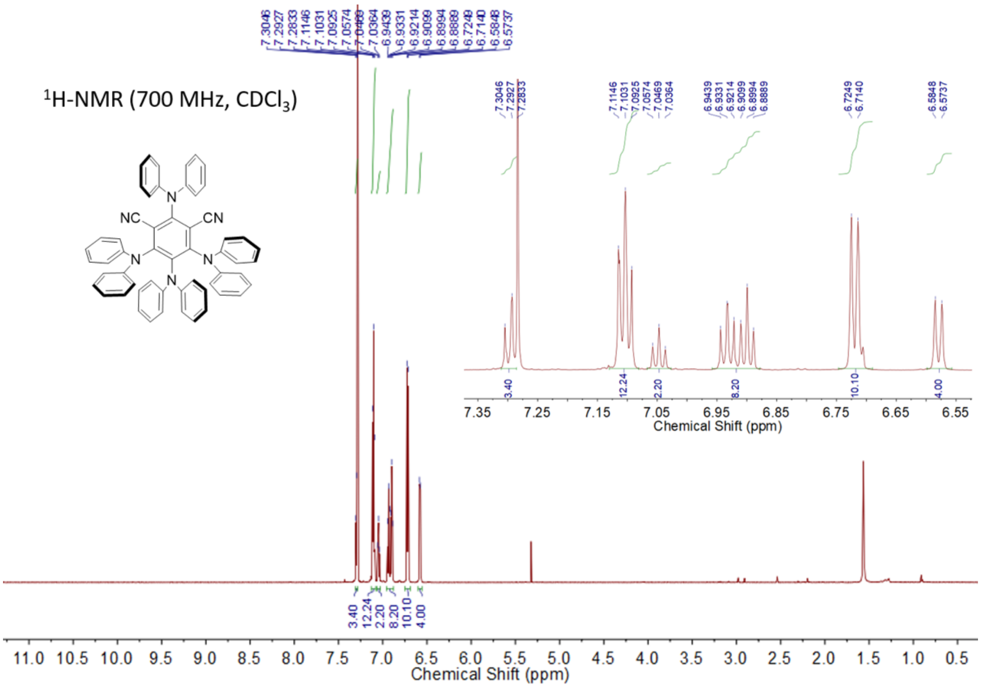

Figure S20. ^{1}H NMR spectrum ($CDCl_3$, 700 MHz) of 2,4,5,6-tetrakis(diphenylamino)isophthalonitrile (4DPAIPN). Residual solvent peaks from CH_2Cl_2 and hexane are visible. ^{1}H NMR (700 MHz, $CDCl_3$): δ = 6.58 (d, J = 7.8 Hz, 4H), 6.70–6.74 (m, 10H), 6.92 (dt, J = 7.7 Hz, 23.4 Hz, 8H), 7.05 (t, J = 7.4 Hz, 2H), 7.08–7.12 (m, 12H), 7.27–7.31 (m, 4H). ^{13}C NMR (176 MHz, $CDCl_3$): δ = 113.0, 113.2, 121.1, 122.6, 122.6, 122.9, 124.0, 124.2, 127.6, 128.6, 129.4, 140.3, 143.2, 144.7, 145.5, 151.7, 154.2.

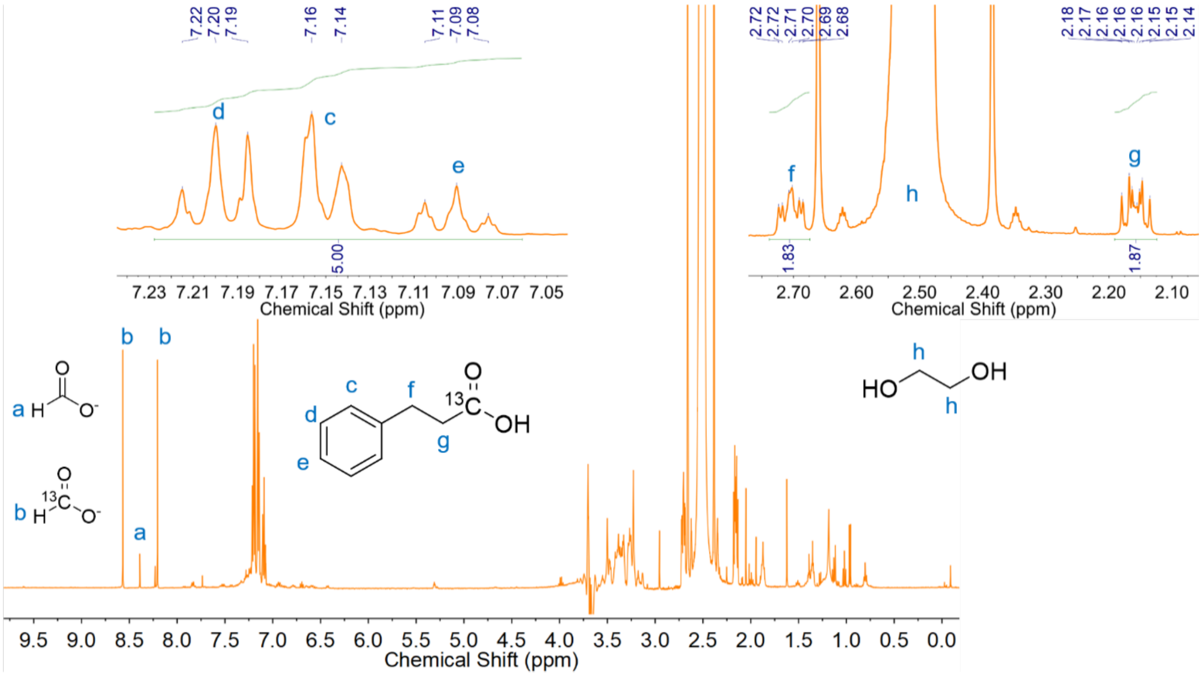

Figure S21. ^{1}H NMR spectrum (d^6-DMSO, 500 MHz) of the solution recovered after the photocatalytic hydrocarboxylation reaction between styrene and an aqueous solution of formate sourced from the anolyte containing ethylene glycol-$^{13}C_2$.

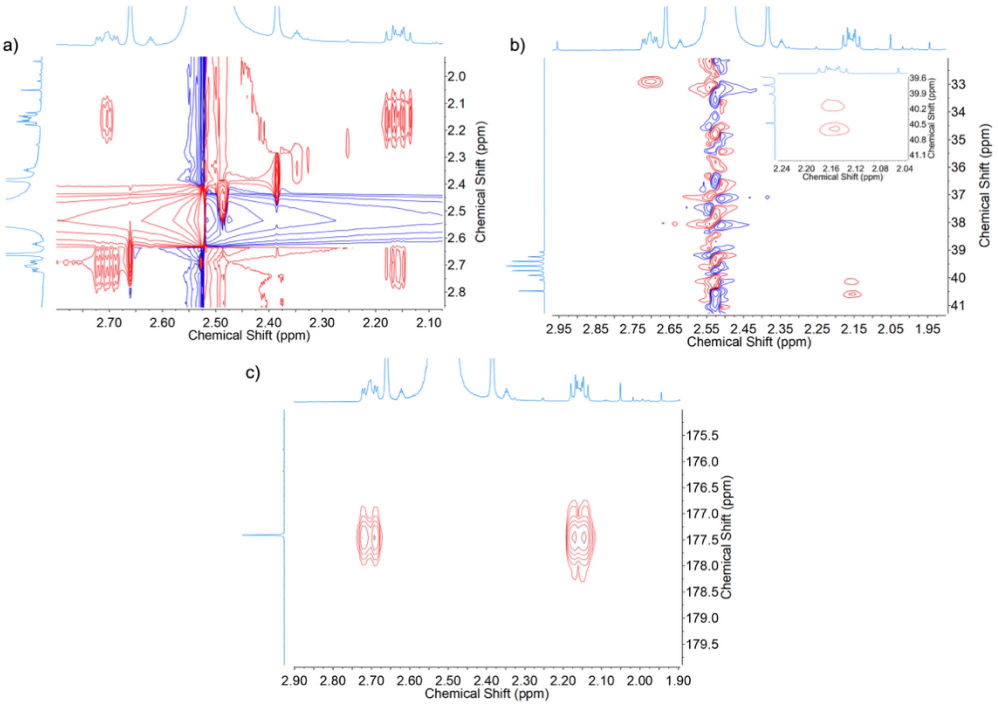

Figure S22. 2D NMR spectra of the solution recovered after the photocatalytic hydrocarboxylation reaction between styrene and an aqueous solution of formate sourced from the anolyte containing ethylene glycol-$^{13}C_2$ (d^6-DMSO, 500 MHz). a) Total correlation spectroscopy (TOCSY) showing the correlation between the protons at 2.70 ppm and 2.17 ppm, assigned to aliphatic protons on C_3 and C_2, respectively. b) High-resolution Heteronuclear Single Quantum Coherence (HSQC). Inset: detail of the signal at (2.17 ppm, 40.4 ppm), showing the splitting along the carbon dimension due to ^{13}C-^{13}C coupling. c) Heteronuclear Multiple Bond Correlation (HMBC), showing the correlation between the ^1H NMR signals of the aliphatic protons on C_2 and C_3 and the ^{13}C NMR signal of the ^{13}C-labelled C_1.

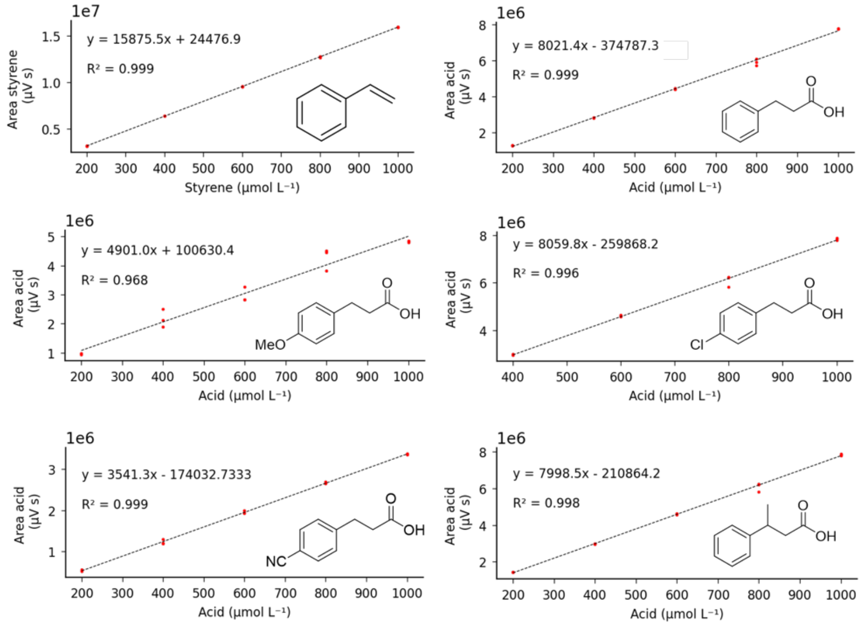

Figure S23. Calibration curves of analytes quantified by HPLC in this work. All curves were extrapolated as a linear fitting of a set of 3 data points for each concentration.

Supporting References

[1] E. Edwardes Moore, V. Andrei, A. R. Oliveira, A. M. Coito, I. A. C. Pereira, E. Reisner, *Angew. Chem. Int. Ed.* **2021**, *60*, 26303-26307.

[2] A. R. Oliveira, C. Mota, C. Mourato, R. M. Domingos, M. F. A. Santos, D. Gesto, B. Guigliarelli, T. Santos-Silva, M. J. Romão, I. A. Cardoso Pereira, *ACS Catal.* **2020**, *10*, 3844-3856.

[3] S. Bhattacharjee, M. Rahaman, V. Andrei, M. Miller, S. Rodríguez-Jiménez, E. Lam, C. Pornrungroj, E. Reisner, *Nat. Synth.* **2023**, *2*, 182-192.

[4] T. Uekert, F. Dorchies, C. M. Pichler, E. Reisner, *Green Chem.* **2020**, *22*, 3262-3271.

[5] J. R. C. Junqueira, D. Das, A. Cathrin Brix, S. Dieckhofer, J. Weidner, X. Wang, J. Shi, W. Schuhmann, *ChemSusChem* **2023**, *16*, e202202349.

[6] S. N. Alektiar, Z. K. Wickens, *J. Am. Chem. Soc.* **2021**, *143*, 13022-13028.

[7] W. L. Chameides, *J. Geophys. Res.* **1984**, *89*, 4739-4755.

[8] E. Edwardes Moore, S. J. Cobb, A. M. Coito, A. R. Oliveira, I. A. C. Pereira, E. Reisner, *Proc. Natl. Acad. Sci.* **2022**, *119*, e2114097119.

[9] I. Bashir, J. D. McGettrick, M. F. Kühnel, B. Sarfraz, S. N. Arshad, A. Rauf, *ACS Sustain. Chem. Eng.* **2024**, *12*, 4795-4802.

[10] Y. Pei, Z. Pi, H. Zhong, J. Cheng, F. Jin, *J. Mater. Chem. A* **2022**, *10*, 1309-1319.

[11] J. Wang, X. Li, M. Wang, T. Zhang, X. Chai, J. Lu, T. Wang, Y. Zhao, D. Ma, *ACS Catal.* **2022**, *12*, 6722-6728.

[12] S. K. Kilaparthi, A. Addad, A. Barras, S. Szunerits, R. Boukherroub, *J. Mater. Chem. A* **2023**, *11*, 26075-26085.

[13] F. Ma, Z. Li, R. Hu, Z. Wang, J. Wang, J. Li, Y. Nie, Z. Zheng, X. Jiang, *ACS Catal.* **2023**, *13*, 14163-14172.

[14] P.-F. Sui, M.-N. Zhu, M.-R. Gao, Y.-C. Wang, R. Feng, X. Wang, S. Liu, J.-L. Luo, *Appl. Catal., B* **2025**, *373*, 125355.

[15] J. Qiu, Z. Jing, D. Zhan, J. Peng, *Chem. Eng. J.* **2025**, *519*, 165447.

[16] S. Hao, M. Cong, Z. Han, H. Xu, T. Liu, M. Guo, X. Ding, Y. Gao, *Chem. Eng. J.* **2024**, *498*, 155106.

[17] H. Liu, Z. Wang, Y. He, X. Hu, L. Liu, *Appl. Catal., B* **2025**, *361*, 124667.

[18] S. K. Kuk, K. Gopinath, R. K. Singh, T.-D. Kim, Y. Lee, W. S. Choi, J.-K. Lee, C. B. Park, *ACS Catal.* **2019**, *9*, 5584-5589.

[19] J. Szczesny, A. Ruff, A. R. Oliveira, M. Pita, I. A. C. Pereira, A. L. De Lacey, W. Schuhmann, *ACS Energy Lett.* **2020**, *5*, 321-327.

[20] Y. Liu, C. W. S. Yeung, E. Reisner, *Energy Environ. Sci.* **2025**.

[21] T. E. Rosser, M. A. Gross, Y.-H. Lai, E. Reisner, *Chem. Sci.* **2016**, *7*, 4024-4035.

[22] C. Chinglenthoiba, G. Mahadevan, J. Zuo, T. Prathyumnan, S. Valiyaveettil, *Nanomater.* **2024**, *14*, 257.
